# Constructing custom thermodynamics using deep learning

**DOI:** 10.1038/s43588-023-00581-5

**Published:** 2023-12-29

**Authors:** Xiaoli Chen, Beatrice W. Soh, Zi-En Ooi, Eleonore Vissol-Gaudin, Haijun Yu, Kostya S. Novoselov, Kedar Hippalgaonkar, Qianxiao Li

**Affiliations:** 1 Department of Mathematics, National University of Singapore, Singapore, Singapore; 2 Institute for Functional Intelligent Materials, National University of Singapore, Singapore, Singapore; 3 Institute of Materials Research and Engineering, A*STAR (Agency for Science), Singapore, Singapore; 4 School of Materials Science and Engineering, Nanyang Technological University, Singapore, Singapore; 5 LSEC and ICMSEC, Academy of Mathematics and Systems Science, Chinese Academy of Sciences, Beijing, China; 6 School of Mathematical Sciences, University of Chinese Academy of Sciences, Beijing, China

**Keywords:** Computational science, Nonlinear phenomena

## Abstract

One of the most exciting applications of artificial intelligence is automated scientific discovery based on previously amassed data, coupled with restrictions provided by known physical principles, including symmetries and conservation laws. Such automated hypothesis creation and verification can assist scientists in studying complex phenomena, where traditional physical intuition may fail. Here we develop a platform based on a generalized Onsager principle to learn macroscopic dynamical descriptions of arbitrary stochastic dissipative systems directly from observations of their microscopic trajectories. Our method simultaneously constructs reduced thermodynamic coordinates and interprets the dynamics on these coordinates. We demonstrate its effectiveness by studying theoretically and validating experimentally the stretching of long polymer chains in an externally applied field. Specifically, we learn three interpretable thermodynamic coordinates and build a dynamical landscape of polymer stretching, including the identification of stable and transition states and the control of the stretching rate. Our general methodology can be used to address a wide range of scientific and technological applications.

## Abstract (web summary)

The authors develop a general method that combines machine learning and physics to construct macroscopic dynamics directly from microscopic observations, leading to an intuitive understanding of polymer stretching in elongational flow.

## Main

The modern scientific method adopts a universal approach that ensures stable and non-conflicting progression of our understanding of nature: new theories need to be hypothesized and tested on previously amassed data, be compatible with the basic scientific principles and be verifiable by experiments. Unfortunately, there is no general algorithmic recipe to do so in complex systems to facilitate discovery. Hence, up to now, only the most basic physical phenomena—often static, in equilibrium—are described by an intuitive set of equations. Many dynamic, non-equilibrium phenomena, which determine functionality in biology, soft-condensed matter and chemistry, are instead described via very approximate, empirical laws. The advancement of artificial intelligence and machine learning gives rise to the possibility of a data-driven solution to this challenge^1,2^.

In this paper, we develop Stochastic OnsagerNet (S-OnsagerNet), an artificial intelligence platform that can discover an interpretable and closed thermodynamic description of an arbitrary stochastic dissipative dynamical system directly from observations of microscopic trajectories. There are essentially two types of approach to understand and predict the behavior of dynamical processes from data—unstructured and structured. Unstructured approaches parameterize dynamical equations by a generic set of building blocks, such as fixed polynomials^3^, trainable feature maps or kernels^4,5^, and determines the associated parameters that best fit the observations. Physical insights can be incorporated as regularizers in the fitting process^6^. Their generality comes at a cost of long-time predictive accuracy, stability^7^ and, more importantly, interpretability. This is addressed by the class of structured approaches, where physical insights directly guide the design of model architectures. Our approach belongs to this latter category. Previous work in this direction includes models based on Hamiltonian or symplectic dynamics^8,9^, Poisson systems^10^ and quasi-potentials^11^. However, so far, there lacks a general structured approach to model dissipative, non-equilibrium and noisy dynamics that often arise in soft matter, biophysics and other applications. Our methodology based on the classical Onsager principle^12,13^ is tailored to such problems.

Macroscopic thermodynamic descriptions of physical systems are highly sought after for the insights they provide. A prototypical example is the ideal-gas law as a macroscopic description of non-interacting gas systems. These systems guide the design of verification experiments and provide principled ways to manipulate macroscopic behavior. For a general complex dynamical system, however, constructing an intuitive thermodynamic description that enables subsequent analysis and control is a daunting task. Our approach addresses this challenge as follows. For a given microscopic dynamics, we learn a macroscopic thermodynamic description via the simultaneous construction of low-dimensional closure coordinates—ensured to be partially interpretable—and a time evolution law on these coordinates. Unlike general artificial intelligence approaches, our platform intrinsically limits the search to physically relevant evolution laws. In particular, we ensure compatibility with existing scientific knowledge by constructing our neural network architecture based on a generalized Onsager principle.

We demonstrate our method by learning the stretching dynamics of polymer chains containing up to 900 degrees of freedom, condensing it into a thermodynamic description involving only three macroscopic coordinates that governs polymer stretching dynamics in both computational and experimental data. We build an energy landscape of the macroscopic evolution, revealing the presence of stable and transition states. This can be viewed as a dynamic equation of state. Mastering such an equation allows the design of verification computational experiments, including the interpretation of the thermodynamic coordinates and the control of the stretching rate of the polymers. We extend this further to conduct single-molecule DNA stretching experiments and show that our thermodynamic description can be used to distinguish fast and slow stretching polymers, much beyond current human-labeling capabilities. Furthermore, the predicted fluctuation correlations derived from the free-energy landscape agree with experimental data.

Constructing low-dimensional physical models from high-dimensional dynamical data is an active area of research. Data-driven modeling of dynamical processes based on the Onsager’s principle was proposed in ref. ^7^ to study Rayleigh–Bénard convection. Reference ^14^ combined encoder–decoders and manifold learning to construct latent dynamical models directly from video data, including those of reaction-diffusion processes and pendulum motions. Here we make several advancements in terms of methodology and applications. First, unlike the deterministic models considered previously, we explicitly capture stochastic fluctuations—an important element of non-equilibrium processes at finite temperatures. In fact, the non-trivial heterogeneity of polymer stretching dynamics studied in this paper is directly caused by thermal fluctuations. Developing our method in the stochastic setting requires extensions of model reduction theory and training algorithms (see ‘Theoretical results and model implementation’ in Methods). Second, and more importantly, we go beyond dimensionality reduction^7,14,15^ and solve a closure problem: given a priori fixed macroscopic variables of interest (for example, polymer extension), we construct both the closure coordinates sufficient to govern the evolution of these macroscopic variables, and the dynamics that describes this evolution. Compared with more flexible parameterizations of reduced dynamics^14^, our approach inherently limits the search space to those satisfying a generalized Onsager principle, which sacrifices complete generality but facilitates physical interpretation of the closure coordinates and the dynamical landscape.

## Results

We now describe our approach. The most complete description of a complex, multi-component system is the coordinates of all the components as a function of time *t* (***X***(*t*)). For the ideal gas, it would be the positions and momenta of all molecules and for a magnetic system—the spin state of each atom. An alternative to this expensive microscopic modeling approach is a thermodynamic one, where the full description is replaced by some macroscopic coordinates (Z*(*t*), with dimensionality much smaller than ***X***(*t*)). This can be the pressure of an ideal gas or the magnetization of a magnetic system. The thermodynamic approach links these macroscopic coordinates to other macroscopic coordinates or closure variables ($\hat{{\boldsymbol{Z}}}(t)$), and external parameters (volume and temperature for ideal gas, magnetic field and temperature for magnetic systems) via an equation of state.

**Fig. 1:**
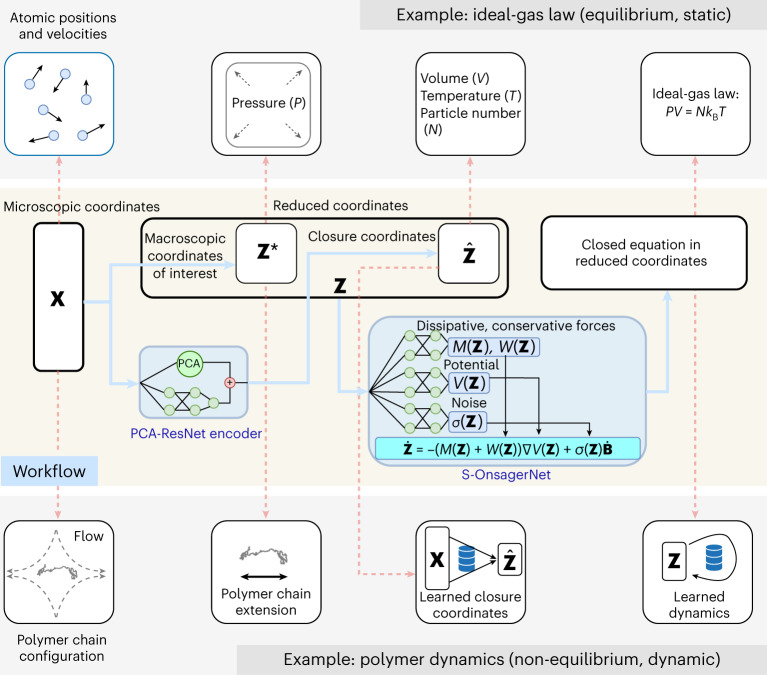
Overall workflow of the proposed approach. Given a complex system described by ***X***, the goal is to model the behavior of macroscopic coordinates of interest Z*. We construct closure coordinates $\hat{{\boldsymbol{Z}}}$ and closed (dynamical) equation on the combined reduced coordinates ${\boldsymbol{Z}}=({\boldsymbol{{Z}}}^{* },\hat{\boldsymbol{{Z}}})$. The classical ideal-gas law is an illustration of this process (top). For general non-equilibrium, dynamic systems (bottom), carrying out this workflow from theoretical analysis is challenging. Our machine learning method (middle) addresses this by simultaneously constructing the closure coordinates using PCA-ResNet (Methods), and governing equations on reduced coordinates using the S-OnsagerNet with drift term −(*M*(***Z***) + *W*(***Z***))∇*V*(***Z***) and noise term *σ*(***Z***) (see equation (2)).

We propose a generic approach of building such custom thermodynamics for an arbitrary stochastic, dissipative dynamical system from data. We are given macroscopic coordinates Z* whose dynamics we wish to model. For polymer dynamics this can be a single variable—the extension of the polymer chain (Fig. 1). Then, we learn a set of closure variables $\hat{{\boldsymbol{Z}}}(t)$ and simultaneously an evolution law on the combined thermodynamic coordinates ${\boldsymbol{Z}}(t)=({{\boldsymbol{Z}}}^{* }(t),\hat{{\boldsymbol{Z}}}(t))$ that enables scientific understanding, experimental verification and control. The evolution equation is a generalization of the classical Onsager principle^12,13^ that has been successfully applied to model a variety of non-equilibrium phenomena, including phase separation kinetics, gel dynamics and molecular modeling^16,17^. It posits a time evolution law

1 $$\begin{array}{rc}\dot{{\boldsymbol{Z}}}(t)&=-M\nabla V({\boldsymbol{Z}}(t)),\end{array}$$

for a given set of coordinates ***Z***(*t*), where the dot denotes time derivative, the symmetric positive semi-definite matrix *M* models energy dissipation and *V* is a generalized potential. A limitation of the Onsager principle is its inability to capture dynamics far from equilibrium, or with substantial stochastic behavior. To this end, we propose an extension in the form of a generalized stochastic Onsager principle

2 $$\dot{\boldsymbol{Z}}(t)=-[M({\boldsymbol{Z}}(t))+W({\boldsymbol{Z}}(t))]\nabla V({\boldsymbol{Z}}(t))+\sigma ({\boldsymbol{Z}}(t)){\dot{\boldsymbol{B}}}(t),$$

where *M*(⋅) and *W*(⋅) are now functions of the reduced coordinates ***Z*** that output *d* × *d* matrices. *M*(⋅) is symmetric positive semi-definite to conform to stability requirements and Onsager’s reciprocal relations^12^, while *W*(⋅) is anti-symmetric and models conservative forces. The diffusion matrix *σ*(⋅) together with the white noise process $\dot{{\boldsymbol{B}}}(t)$ models the thermal fluctuations in the system. Equation (2) forms the basis of our dynamical model in reduced coordinates. We note that alternative generalizations of the Onsager principle have been proposed using large deviations theory^18^, but their forms are more complex and hence less amenable to computations. It can be shown that our model has long-time stability through energy dissipation up to the order of thermal fluctuations (Theorem 2) and the flexibility to represent many physical stochastic processes, including Langevin and generalized Poisson dynamics (see ‘Theoretical results’ in Methods). Our method departs from classical modeling paradigms, where the unknown equation parameters are few and can be fitted from few experiments. Instead, the unknowns here are functions *M*, *W*, *V* and *σ*. We leverage machine learning and represent these functions as trainable deep neural networks, while preserving the required physical constraints (for example, symmetric positive definiteness of *M*). Simultaneously, we generate a set of the closure coordinates by another deep neural network, which combines approximation flexibility of residual networks and approximate feature orthogonality through principal component analysis (PCA). This is to be contrasted with generic coarse-graining methods based on volume averages^19^, in that we seek a very small set of closure coordinates that are sufficient to describe the motion of the macroscopic states of interest. Our learning-based approach to discover hidden coordinates shares some similarity with recently proposed machine learning-based coarse-graining methods in molecular simulations^15,20^ but here we work with a closure problem, thus we may end up with macroscopic dynamics of substantially lower dimensions. We perform end-to-end training of the combined architecture on large-scale microscopic trajectory data to simultaneously learn the reduced coordinates and their dynamics. The overall workflow for creating custom thermodynamics, which we call S-OnsagerNet, is summarized in Fig. 1. Detailed network architectures and training algorithms are given in Methods.

### Training and prediction of polymer stretching dynamics

We first demonstrate our approach by modeling the temporal evolution of polymer extension under elongational forces, which has long been of interest to the polymer physics community^21–24^. Hallmark experimental^25,26^ and computational studies^27–29^ in elongational rheology of dilute polymers have examined the deformation of single DNA molecules in planar elongational flows and revealed the highly heterogeneous stretching dynamics among identical polymer chains. Due to the complex interactions within and stochastic nature of the system, it is challenging to identify macroscopic descriptors of the polymer chain (closure coordinates) and governing equations on these descriptors that are sufficient to determine the outcome of the stretching dynamics. Yet, such a thermodynamic description is essential for understanding the origins of unfolding heterogeneity and paves the way to make desired modifications to the unfolding dynamics. Thus, our data-driven method offers a promising alternative to achieve this goal.

**Fig. 2:**
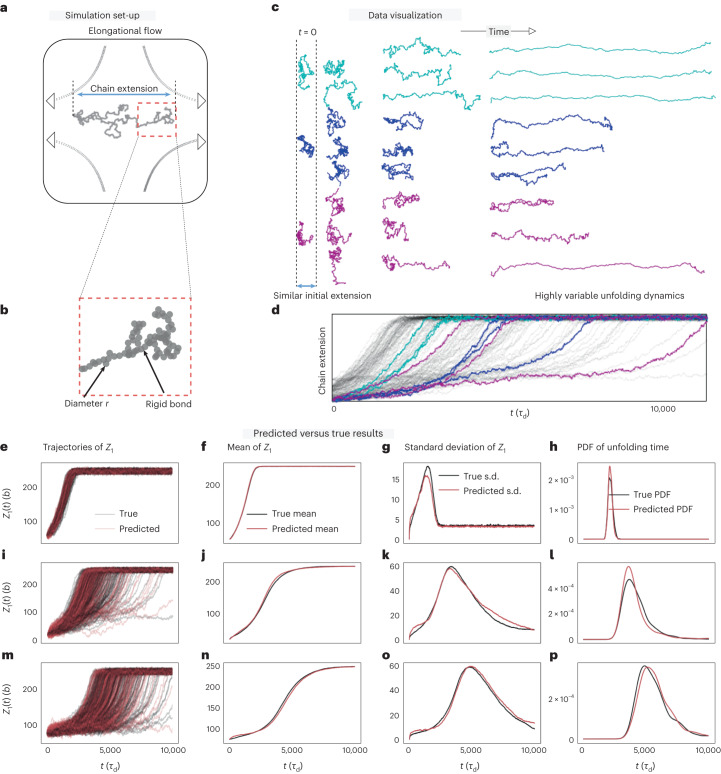
Simulation set-up, data visualization, and predicted versus true stretching trajectories and their statistics. **a**,**b**, The polymer chain is represented by a bead-rod model with bead diameter *r* (in units of *b* = 10 nm) and rigid bonds, subjected to hydrodynamic and Brownian forces. **b** shows a magnified portion of the polymer chain shown in **a**. **c**,**d**, The statistics of chain extension projected along the elongational axis are recorded. **c** shows the stretching polymers at different time instants and d depicts the extensions as a function of time for each trajectory. Times are reported in units of the characteristic rod diffusion time *τ*_d_ (see ‘Data preparation’ in Methods). Different initial conditions (colors) are chosen to have similar initial extension but varying unfolding times. Identical initial configurations also have different unfolding dynamics due to thermal fluctuations. **e**–**p**, S-OnsagerNet can capture this heterogeneity. **e**–**g**, We plot 500 trajectories of polymer extensions (*Z*_1_) from the same initial condition (**e**), together with their mean (**f**) and standard deviation (**g**). **h**, The probability density functions (PDFs) of unfolding times. **i**–**p**, Results for two other unseen initial chain configurations.**i** and **m** shows the extension length as a function of time for the medium and fast trajectories respectively. Similarly, **j** and n show the means, **k** and **o** the standard deviations, and **l** and **p** the probability density functions of unfolding times for the medium and fast trajectories. respectively. Source data

We simulate polymer chain stretching in a planar elongational flow. The polymer chain consists of 300 coarse-grained beads connected by rigid rods, resulting in 900 degrees of freedom if we ignore inertial effects (Fig. 2a,b). Snapshots of the shape of the chains under stretching conditions are shown in Fig. 2c, revealing highly heterogeneous dynamics of the chain extension (Fig. 2c,d), defined as the projected chain length along the elongational axis of the flow. This is our macroscopic coordinate of interest Z*(*t*). Our aim is to model its stochastic evolution and understand the origin of its heterogeneity. We train the S-OnsagerNet on this dataset following the workflow in Fig. 1. The network architecture selection and training procedures are found in Methods. Our approach constructs two closure coordinates in addition to the chain extension Z*(*t*), leading to a three-dimensional dynamical system—following equation (2) with learned functions *M*, *W*, *V* and *σ*—that governs the dynamics of stretching. We have empirically chosen the number of macroscopic coordinates: using more than three did not substantially improve predictive accuracy, whereas a two-variable system has modeling limitations due to physical symmetry. The detailed selection procedure of the reduced coordinate dimension is discussed in ‘Polymer dynamics analysis’ in Methods.

In Fig. 2e–m, we test the trained S-OnsagerNet on three unseen, different and representative initial polymer configurations. The selected chains start with similar extension lengths, but subsequently stretch at vastly different rates. Figure 2e–g,i–k,m–p shows that the true statistics (black) can be accurately predicted (red). Moreover, the distributions of the time taken to reach a reference extension length are successfully captured (Fig. 2h,i,m).

### Interpreting learned closure coordinates

**Fig. 3:**
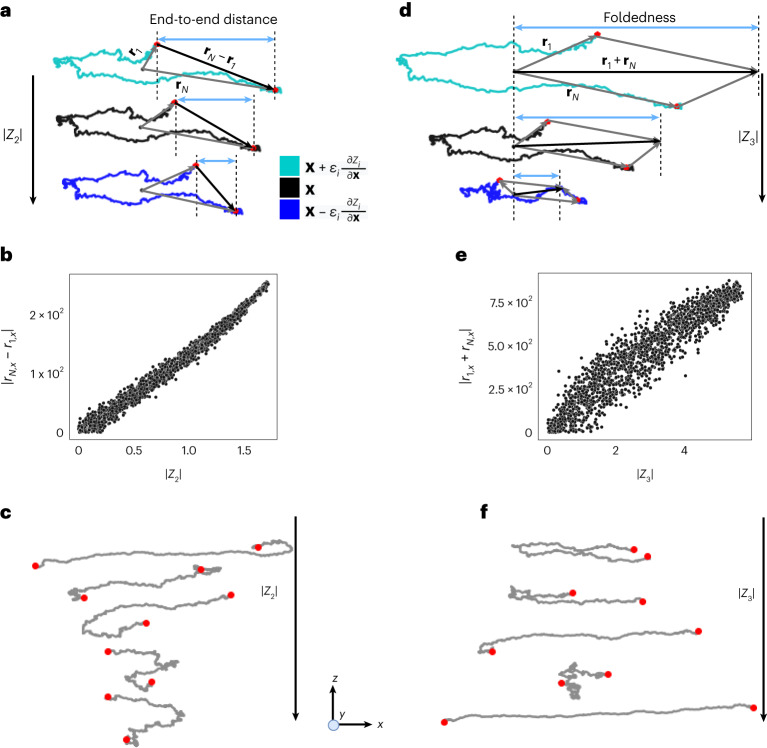
Physical interpretation of learned closure coordinates. **a**, Perturbation of the polymer chain ${\boldsymbol{X}}\pm {\varepsilon }_{2}\frac{\partial {Z}_{2}}{\partial {\boldsymbol{X}}}$ from a given configuration, ${\varepsilon }_{2}=100{/}\left\Vert \frac{\partial {Z}_{2}}{\partial {\boldsymbol{X}}}\right\Vert_{2}$. **b**, Plot of projected end-to-end distance |*r*_*N*,*x*_ − *r*_1,*x*_| as a function of |*Z*_2_| for the training data. **c**, Configurations of different polymer chains with decreasing |*Z*_2_| values. As |*Z*_2_| decreases, the projected distance between the chain ends decreases. **d**, Perturbation of the polymer chain ${\boldsymbol{X}}\pm {\varepsilon }_{3}\frac{\partial {Z}_{3}}{\partial {\boldsymbol{X}}}$ from a given configuration, ${\varepsilon }_{3}=260{/}\left\Vert \frac{\partial {Z}_{3}}{\partial {\boldsymbol{X}}}\right\Vert_{2}$. **e**, Plot of degree of foldedness |*r*_1,*x*_ + *r*_*N*,*x*_| as a function of |*Z*_3_| for the training data. **f**, Configurations of different polymer chains with decreasing |*Z*_3_| values. As |*Z*_3_| decreases, the chains tend towards a more stretched state. Source data

Having shown that only two closure coordinates $\hat{{\boldsymbol{Z}}}=({Z}_{2},{Z}_{3})$ are required to characterize the stochastic evolution of the extension length Z* = *Z*_1_, it is natural to probe the meaning of these discovered coordinates. Here we utilize an intrinsic property of neural networks—it represents the nonlinear reduction functions ${\boldsymbol{X}} \mapsto {\boldsymbol{Z}}$ as differentiable maps, as though we have learned their analytical forms. We compute via automatic differentiation the perturbations on a generic microscopic configuration *X* in the directions of ±∂*Z*_2_/∂***X*** and ±∂*Z*_3_/∂***X***, corresponding to conformations with steepest changes in *Z*_2_ and *Z*_3_, respectively. The resulting conformational changes suggest physical interpretation of these coordinates. For example, from Fig. 3a, we observe that perturbations in the direction of ∂*Z*_2_/∂***X*** tend to change the end-to-end distance in the elongational axis, or distance between the first and the last bead in the polymer chain along the elongational axis. We confirm this hypothesis by visualizing the correlation of the end-to-end distance and the magnitude of *Z*_2_ in Fig. 3b,c. A similar analysis reveals the correlation between *Z*_3_ and a degree of foldedness of the chain in the elongational axis of the flow (Fig. 3d–f).

### Free-energy landscape

**Fig. 4:**
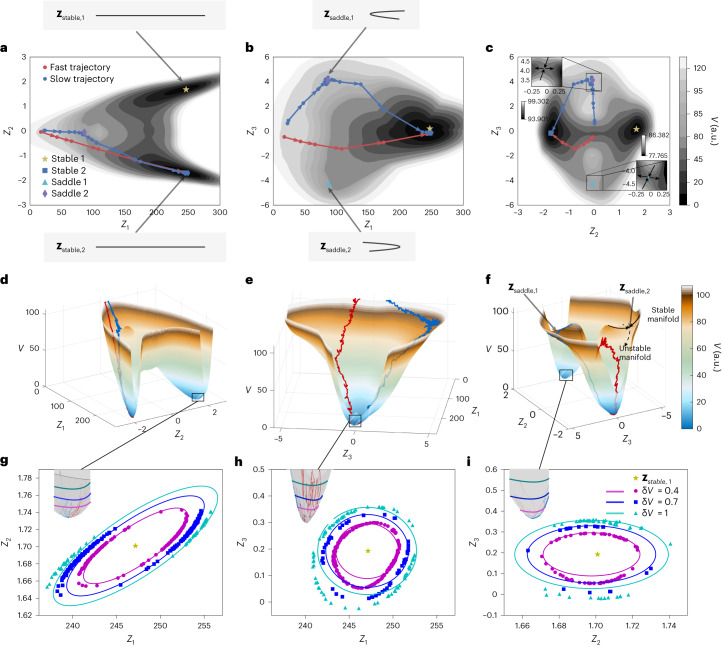
Learned potential energy landscape. **a**–**f**, We plot *V* projected onto the *Z*_1_–*Z*_2_ (**a**,**d**), *Z*_1_–*Z*_3_ (**b**,**e**) and *Z*_2_–*Z*_3_ (**c**,**f**) planes. **a–c** are contour plots and **d**–**f** are surface plots to visualize the landscape. Insets: stable and unstable directions of the saddle points, corresponding to positive (top left inset) and negative (bottom right inset) *Z*_3_ values. Projection is computed via minimization (for example, $V({Z}_{1},{Z}_{2})=\mathop{\min }\limits_{{Z}_{3}}V({Z}_{1},{Z}_{2},{Z}_{3})$), which at low temperatures closely approximates marginalizing with respect to the Boltzmann distribution. The stable and saddle points are marked on the energy landscape, and their corresponding reconstructed fully extended and folded states are shown. A pair of each exists due to reflection symmetry in the flow direction. Example ‘fast’ (red) and ‘slow’ (blue) trajectories from the training dataset are overlaid on the landscape. The fast trajectory avoids the saddle points and goes directly towards a stable minimum, whereas the slow trajectory gets trapped for long times near saddle 2, before finally escaping through its unstable manifold. For **b** and **e**, the stable manifolds of the saddles closely align with *Z*_2_, and hence are not visible due to minimization (marginalization). **g**–**i**, Scatterplots in the *Z*_1_–*Z*_2_ (**g**), *Z*_1_–*Z*_3_ (**h**) and *Z*_2_–*Z*_3_ (**i**) planes, together with predicted isotherms (solid lines) capturing typical fluctuations around a fully stretched state ***Z***_stable,1_. Insets: magnified views of the fluctuating trajectories around the stretched state over the energy landscape. Source data

The constructed potential *V* can be interpreted as a generalized free energy, allowing us to gain important insights into the dynamical landscape. The local minima of *V* represent stable or metastable states, while the saddle points correspond to transition states. The differentiable representation of *V* enables us to probe this landscape. Figure 4 shows two-dimensional projections of the three-dimensional free energy *V*(*Z*_1_, *Z*_2_, *Z*_3_). We identify the critical points of *V* by solving ∇*V*(***Z***) = 0 using the Broyden–Fletcher–Goldfarb–Shanno (BFGS) method. We found two stable fixed points and two saddle points of interest marked in Fig. 4. Using a simultaneously trained PCA-ResNet decoder, we can reconstruct the macroscopic spatial coordinates of the polymer chain at the critical points to identify their physical origin. Up to reflection symmetry in the elongational axis, the stable points correspond to fully stretched states, whereas the saddle points refer to completely folded states. The origin of the heterogeneity in unfolding times is now clear: a rapidly stretching polymer is the one that avoids the saddle point and goes directly to a stable minimum, whereas a slowly stretching chain gets trapped around the stable manifold (attractive part) of the saddle point for a long time, before finally escaping through the unstable manifold (repulsive part) of the saddle towards the stable minimum (Supplementary Video 1). We confirm this by overlaying a fast and a slow unfolding trajectory with the potential landscape in Fig. 4c,f. Despite the similarity between the initial chain configurations, as demonstrated by the proximity between the initial points on the potential energy landscape, the chains show different stretching behaviors that can be rationalized by the constructed potential.

Moreover, a Taylor expansion via automatic differentiation of the learned *V*(***Z***) captures the leading-order fluctuations near a stable stretched state. We denote by δ*V* a typical energy fluctuation around the stretched state (proportional to temperature). Then, ignoring small terms we find

3 $$\updelta V\approx {a}_{1}\,{(\updelta {Z}_{1}-{a}_{4}\updelta {Z}_{2})}^{2}+{a}_{2}\,\updelta {Z}_{2}^{2}+{a}_{3}\,\updelta {Z}_{3}^{2},$$

where *a*_1_ = 153.1, *a*_2_ = 205.5, *a*_3_ = 36.96, *a*_4_ = 1.54 and $\updelta {Z}_{i}={Z}_{i}-{[{Z}_{{{{\rm{stable}}}},1}]}_{i}$ is the fluctuations in the thermodynamic variables. Note that the coefficients *a*_*j*_ implicitly depend on the strength of the flow. Equation (3) is an effective equation of state, from which we observe the positive correlation of *Z*_1_ and *Z*_2_. The physical interpretation is that near the stretched state, the chain extension and the end-to-end distance tend to change simultaneously (see Fig. 4g–i and ‘Polymer dynamics analysis’ in Methods).

### Controlling polymer stretching dynamics

**Fig. 5:**
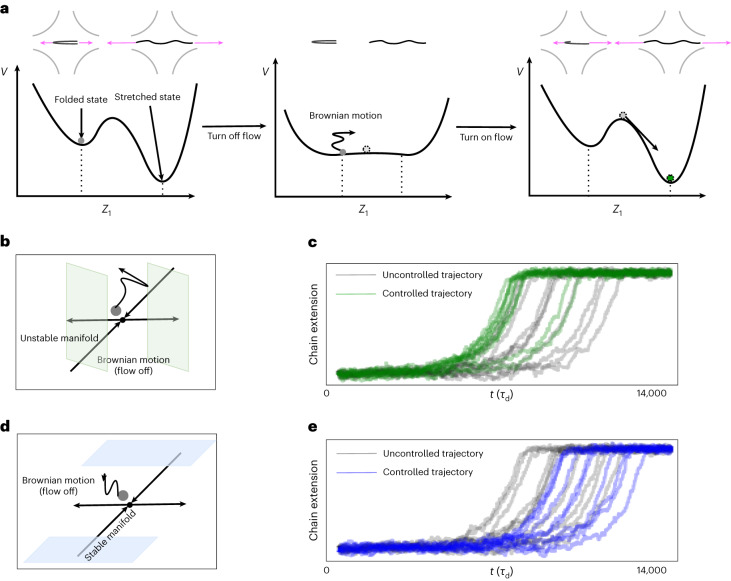
Data-driven control of the stretching dynamics. **a**, Control protocol to speed up unfolding. Left: projected along the extension direction, the polymer must overcome energy barriers to transition from the folded to the stretched state. Middle: in the control protocol, the flow is turned off if the reduced coordinate of the polymer is near the saddle point corresponding to the folded state, and the polymer drifts under the Brownian motion. Right: then the flow is turned back on if the reduced coordinates of the polymer becomes sufficiently aligned (green shaded region in **b**) with the unstable manifold of the saddle or when the the equilibration time reaches 100 *τ*_d_. Without any control, a folded state near the saddle point will unfold slowly (gray lines in **c**); with control, the chains unfold more rapidly (green lines in **c**). **b**, Schematic of the control protocol near the saddle point. **c**, Controlled and uncontrolled chain extension dynamics. For the slowest 10 trajectories shown, their mean unfolding time was reduced by 14.14%. **d**,**e**, Reversed control protocol (**d**) to slow down unfolding by turning on the flow when the reduced coordinates become aligned with the stable manifold instead (blue shaded region in **d**). **e** shows that the mean unfolding time increased by 14.96% (blue lines in **e**). Source data

Further, understanding the laws of the custom thermodynamics of polymer chain folding allows us to interact with the dynamics by designing controls over the polymer environment to initiate desired changes in its behavior. To this end, we perform another Taylor expansion of *V*—this time near a saddle point ***Z***_saddle,2_ corresponding to a folded state to give another local equation of state

4 $$\updelta V\approx {b}_{1}\,\updelta {Z}_{1}^{2}-{b}_{2}\,{(\updelta {Z}_{2}-{b}_{4}\updelta {Z}_{3})}^{2}+{b}_{3}\,\updelta {Z}_{3}^{2},$$

where *b*_1_ = 102.96, *b*_2_ = 31.13, *b*_3_ = 24.16 and *b*_4_ = 0.255. Equation (4) suggests that to escape this saddle point leading to polymer unfolding, it is most effective to increase end-to-end distance (*Z*_2_) while decreasing foldedness (*Z*_3_) in a proportional way. This leads to a data-driven control protocol in Fig. 5a,b. We choose the external elongational flow as the only control parameter (in real experiments it corresponds to switching on and off the flow of fluid or the electric field^26^). We start with a polymer configuration near the saddle point of the energy landscape, corresponding to a folded state. Without any intervention, the subsequent unfolding is expected to follow a slow trajectory, staying near the folded state for a long time. From our landscape analysis above, the most effective escape from the saddle point is along its unstable manifold—approximately corresponding to increasing |δ*Z*_2_ − *b*_4_δ*Z*_3_|. Thus, to speed up unfolding, we can design the following control strategy: we turn off the external elongational flow, so that the polymer drifts randomly under Brownian forces around the saddle point. We track its reduced coordinates, and once we observe sufficient alignment with the unstable manifold, we turn on the externally applied elongational flow. We observe in Fig. 5c that this simple control system speeds up the unfolding dynamics substantially. We can also increase the unfolding time by reversing this protocol (Fig. 5d,e). These control strategies based on the learned thermodynamic description have notable advantages over classical model-free control regimes (for example, reinforcement learning), which may require large exploration times or small, finite state spaces^30^.

### Experimental validation

**Fig. 6:**
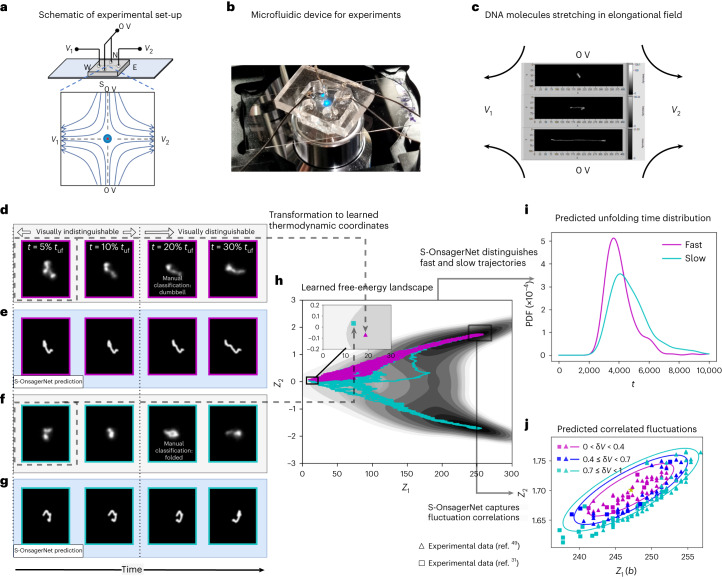
Analysis of experimental data. **a**,**b**, Schematic (**a**) and photograph (**b**) of the experimental set-up, consisting of a microfluidic cross-slot device and platinum electrodes in the reservoirs. Via the electrodes, positive voltage levels *V*_1_ and *V*_2_ are applied to the west and east reservoirs (W and E, respectively), while the north and south reservoirs (N and S, respectively) are kept at 0 V. During trapping, the negatively charged DNA will thus flow from N/S to W/E, until a molecule is trapped at the center of the channel—blue dot in the schematic. **c**, Snapshots of a DNA molecule stretching. **d**,**f**, Processed experimental images at various percentages of the unfolding time (*t*_uf_) of a fast (**d**) and a slow (**f**) trajectory. The selected trajectories have similar initial configurations and are visually indistinguishable in terms of unfolding dynamics. **h**, Learned potential landscape and predicted slow and fast trajectories using S-OnsagerNet with the initial configurations at *t* = 5% *t*_uf_ from **d** and **f**. We note slight differences in the initial *Z*_2_ values only (inset). **e**,**g**, Reconstructed high-dimensional configurations of selected simulated trajectories with similar low-dimensional coordinates as the experimental configurations in **d** (corresponding to **e**) and **f** (corresponding to **g**). The S-OnsagerNet is capable of distinguishing between the manually classified dumbbell and folded states. **i**, Predicted PDFs of unfolding time with initial condition of fast and slow experimental trajectories at *t* = 5% *t*_uf_ using S-OnsagerNet. **j**, Fluctuations in *Z*_1_ and *Z*_2_ around the stable (stretched) state from experimental images. Data were obtained from ref. ^49^ (triangle) and ref. ^31^ (square). Reduced coordinates are constructed according to the procedure outlined in ‘Data preparation’ in Methods. Colors indicate predicted energy levels according to the learned potential. We observe that fluctuations in *Z*_1_ and *Z*_2_ are highly correlated and agree well with that predicted by the effective equation of state.

Remarkably, some qualitative predictions of the constructed thermodynamic description are confirmed by physical experiments. Not only can one show that the constructed dynamical landscape allows for fine-grained classification of stretching behavior on simulation data (see ‘Polymer dynamics analysis’ in Methods), but also we demonstrate in Fig. 6 that this applies directly to physical experiments. Here we perform single-molecule experiments to observe the stretching trajectories of DNA molecules in a planar elongational field (see ‘Data preparation’ in Methods). We select two samples that initially appear similar (Fig. 6e,g), making it impossible to visually distinguish them in terms of stretching behavior. We then cast them into the learned thermodynamic coordinates ***Z***, which when superimposed on the free-energy landscape reveals that the *Z*_2_ coordinates differ subtly, leading to different predicted stretching statistics (Fig. 6i). This substantially improves on human-level labeling, which can only occur much later in the dynamical evolution (Fig. 6h,j). Furthermore, we show in Fig. 6j that the effective equation of state equation (3) that captures the correlations of *Z*_1_ and *Z*_2_ around the stretched state also applies to experimental data from two sources, including the current experiments and previously available data^31^. These results demonstrate the promise of the current approach in enabling physical understanding and control of real polymer dynamics.

### Modeling spatial epidemics

**Extended Data Fig. 1:**
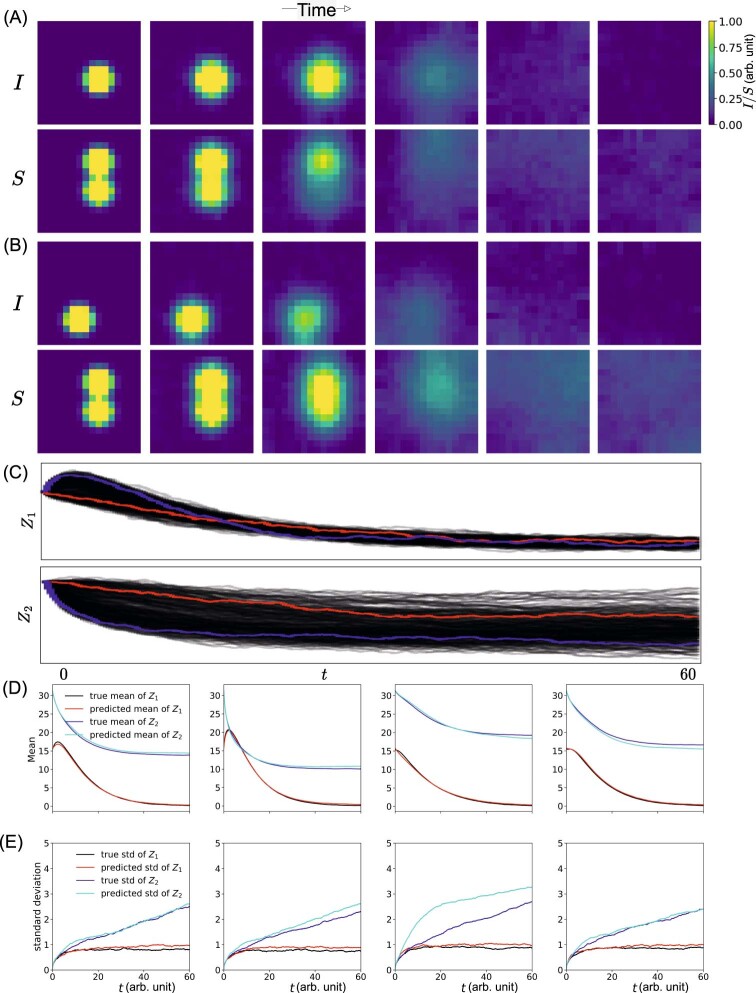
Data visualization and prediction results. A) and (B): Two trajectories depicting the spatial evolution of *I* (infective) and *S* (susceptible) with distinct initial conditions are plotted. They have identical spatial averages initially but differing subsequent evolution. In particular, in (A) the disease spreads (*Z*_1_, the spatial average of *I* increases initially) but in (B) the disease dies out monotonically. (C) Scatter of *Z*_1_ and *Z*_2_ (spatial average of *S*) trajectories, showing a high degree of variability despite identical initial values. Note that there is variability in both the presence of disease spread (*Z*_1_ increasing initially) and the terminal value of *Z*_2_, corresponding to the remaining uninfected population after the epidemic. The blue (resp. red) trajectory corresponds to (A) (resp. (B)). (D, E) True vs predicted statistics using S-OnsagerNet with four different test initial conditions, showing good agreement. Source data

**Extended Data Fig. 2:**
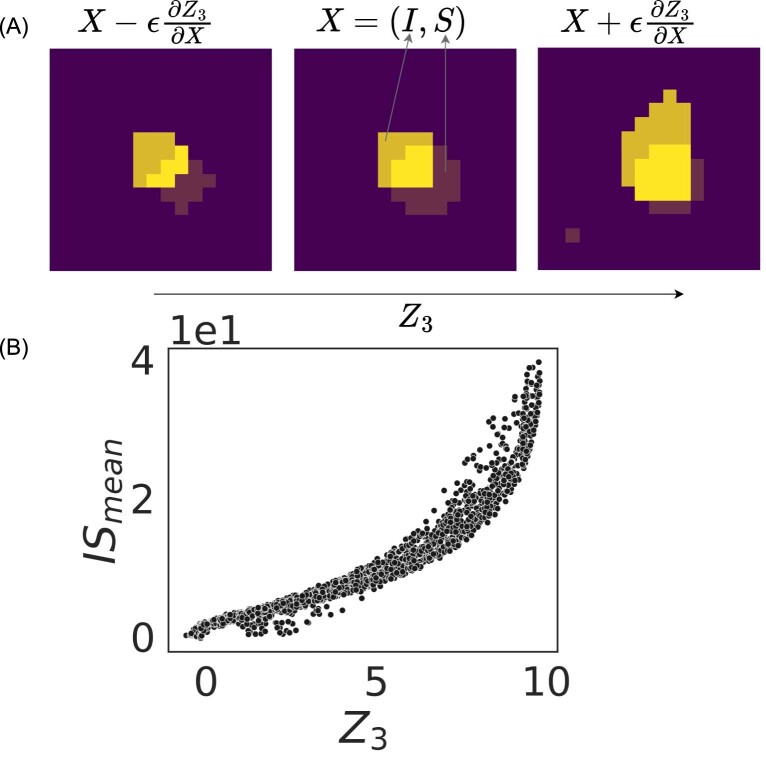
Physical interpretation of learned closure coordinate. (A) Perturbations *X* ± $\epsilon$∂*Z*_3_/∂*X* from a given configuration *X* = (*I*, *S*) with $\epsilon$ = 2 are shown. The are overlapping clusters, in the form of Gaussians centered at (0, 0) for *S* and (-1, 1) for *I*. To illustrate the overlap clearly, we binarize the values as follows: if the value of *I* and *S* is greater than 1, we truncate it to 1; otherwise, if the value is less than 1, we truncate it to 0. Observe from (A) that increasing *Z*_3_ corresponds to increasing spatial overlap of the clusters, and vice versa. We confirm this in (B), where we plot *IS*_*mean*_ (spatial overlap) vs *Z*_3_, showing a positive correlation. Source data

**Extended Data Fig. 3:**
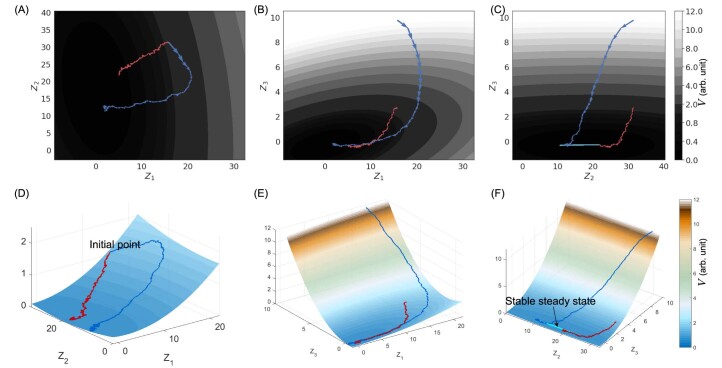
Potential landscape of the SIR model. The learned *V* is projected onto *Z*_1_ − *Z*_2_ (A,D), *Z*_1_ − *Z*_3_ (B, E) and *Z*_2_ − *Z*_3_ (C, F) planes. Projection is computed via minimization (for example $V({Z}_{1},{Z}_{2})=\mathop{\min }\limits_{{Z}_{3}}V({Z}_{1},{Z}_{2},{Z}_{3})$. Example of disease spread (blue) and disease dying out (red) trajectories with the same initial *Z*_1_ and *Z*_2_ from the training dataset are shown. We observe from (B) that *Z*_3_ determines the onset of disease spread and differentiates the two trajectories. Moreover, (C) shows that *Z*_3_ also differentiates the final outcome of the epidemics, where the final *Z*_2_ value depends on the initial *Z*_3_ value, and belongs to a 1D manifold of stable steady states as shown in (F). Source data

To further demonstrate general applicability, we employ our method to derive macroscopic dynamics of spatial epidemics. The classical spatial Susceptible-Infectious-Recovered (SIR) model^32^ governs microscopic evolution of infective and susceptible individuals on a spatial domain (Extended Data Fig. 1a–c). Using these microscopic trajectories, we construct a thermodynamic model that accurately models the evolution of the spatial averages of infective and susceptible individuals (Extended Data Fig. 1d,e) with an additional learned closure coordinate. Moreover, following the same approach as before, we can interpret this coordinate as the spatial overlap of infective and susceptible individuals (Extended Data Fig. 2), thereby rationalizing the dynamical landscape (Extended Data Fig. 3), where this overlap determines the onset and outcome of disease spread. Details are found in ‘Spatial epidemics analysis’ in Methods.

## Discussion

The potential applicability of our method goes beyond polymer and epidemic dynamics, and includes general complex dissipative processes such as protein folding^33^, self-assembly^34,35^ and glassy systems^36,37^. Despite the importance of potential energy landscapes for material systems with functional properties, the challenge in constructing them has limited current approaches to systems with small degrees of freedom and/or requiring judicious selection of system descriptors based on expert knowledge^38^. The method described in this work offers the potential of automating this process, creating pathways towards a multitude of opportunities for understanding and control over various complex systems and their scientific applications.

There are many worthwhile future research directions to further improve the robustness and generality of the proposed method. Here we inherently constrain the search space to macroscopic dynamics that conform to the generalized stochastic Onsager principle. Thus, it naturally has limited ability to model systems that may not readily admit such a description, such as chaotic systems. Moreover, the present model reduction and stability theory require the thermal noise to be small compared with the dissipative and conservative forces. While this is the case for the polymer dynamics studied here, our theory needs to be expanded to handle highly stochastic cases. In terms of training methodology, the current trial-and-error selection of the dimension of the closure variables can be made more systematic, for example, by building on manifold learning approaches^14^. Another potential improvement is the data sampling process. We observed in our numerical experiments that accurate construction of the dynamical landscape requires the trajectory data to sufficiently sample the regions of interest (stable and transition states). An adaptive or active learning algorithm^39,40^ that couples data sampling and S-OnsagerNet training can be developed to improve on the current random sampling strategy. On the scientific problem of polymer dynamics, we have considered motion under only a single stretching force. It is worthwhile to extend our study to varying stretching conditions to build a more comprehensive picture of polymer stretching. More broadly, one may apply our approach to learn macroscopic thermodynamics of other systems of scientific interest.

## Methods

### Theoretical results

In this section, we collect a number of theoretical results concerning the S-OnsagerNet approach. We first show that if a high-dimensional stochastic dynamical system satisfies the generalized stochastic Onsager principle (GSOP), then, any well-behaved reduction into a lower-dimensional system will result in one that obeys approximately the GSOP introduced in equation (2) (Theorem 1). An immediate consequence is that our model reduction approach is theoretically justified for a wide variety of dissipative and conservative systems, including molecular dynamics^41^, stochastic Hamiltonian systems^42^ and the stochastic Lotka–Volterra model^43^. Next, we prove that dynamics described by the GSOP satisfy an energy dissipation law (Theorem 2) and thus our machine learning approach produces stable dynamics at sufficiently low temperatures.

For convenience, we show that there are two equivalent forms of the GSOP. The formulation of the GSOP we use to construct our neural networks is

5 $$\begin{array}{rc}{\mathrm{d}}{\boldsymbol{Z}}(t)&=-(M({\boldsymbol{Z}}(t))+W({\boldsymbol{Z}}(t)))\nabla V({\boldsymbol{Z}}(t)){\mathrm{d}}t+\sigma ({\boldsymbol{Z}}(t)){\mathrm{d}}{\boldsymbol{B}}(t).\end{array}$$

Here, *B* is the standard Brownian motion. Now, assuming *M*(⋅) + *W*(⋅) is invertible, we define

$$\begin{array}{rcl}\tilde{M}({\boldsymbol{Z}}\,)&=&\displaystyle\frac{{(M({\boldsymbol{Z}}\,)+W({\boldsymbol{Z}}\,))}^{-1}+{({(M({\boldsymbol{Z}}\,)+W({\boldsymbol{Z}}\,))}^{-1})}^{T}}{2},\\ \tilde{W}({\boldsymbol{Z}}\,)&=&\displaystyle\frac{{(M({\boldsymbol{Z}}\,)+W({\boldsymbol{Z}}\,))}^{-1}-{({(M({\boldsymbol{Z}}\,)+W({\boldsymbol{Z}}\,))}^{-1})}^{T}}{2},\\ \tilde{\sigma }({\boldsymbol{Z}}\,)&=&{(M({\boldsymbol{Z}}\,)+W({\boldsymbol{Z}}\,))}^{-1}\sigma ({\boldsymbol{Z}}\,).\end{array}$$

Observe that equation (5) can be rewritten in the form

6 $$({\tilde{M}}({\boldsymbol{Z}}(t))+{\tilde{W}}({\boldsymbol{Z}}(t))){\mathrm{d}}{\boldsymbol{Z}}(t)\quad = - \nabla V({\boldsymbol{Z}}(t)){\mathrm{d}}t+{\tilde{\sigma}}({\boldsymbol{Z}}(t)){\mathrm{d}}{\boldsymbol{B}}(t).$$

A similar construction shows that we can also rewrite equation (6) in the form of equation (5) for any $\tilde{M},\tilde{W},\tilde{\sigma }$ assuming similar invertibility conditions. Thus, they are in fact equivalent. While the numerical implementation is based on equation (5), the form equation (6) is also useful, and in the following we refer to both as GSOP.

Now, we demonstrate the general applicability of the GSOP in the context of model reduction. We consider a microscopic (high dimensional) dynamics satisfying a GSOP of the form

7 $$\begin{array}{rc}{\mathrm{d}}{\boldsymbol{X}}(t)&=-\left({M}_{1}({\boldsymbol{X}}(t))+{W}_{1}({\boldsymbol{X}}(t))\right)\nabla {V}_{1}({\boldsymbol{X}}(t)){\mathrm{d}}t+\sqrt{{\epsilon }_{1}}{{{\varSigma }}}_{1}({\boldsymbol{X}}(t)){\mathrm{d}{\boldsymbol{B}}}_{1}(t),\end{array}$$

where ${\boldsymbol{X}}(t)\in {{\mathbb{R}}}^{D},{M}_{1}(\cdot ),{W}_{1}(\cdot )$ are symmetric positive semi-definite and anti-symmetric matrix valued functions respectively, *Σ*_1_(⋅) is the *D* × *p*_1_-dimensional diffusion matrix, ***B***_1_ is a *p*_1_-dimensional Brownian motion and $\epsilon$ is a positive parameter related to temperature (for example, $\epsilon_1$ ∝ *k*_B_*T* with *k*_B_ being Boltzmann’s constant and *T* the temperature).

We now show that many high-dimensional systems of physical interest indeed satisfy a version of GSOP. We first consider the well-known Langevin dynamics, which has been used to model many stochastic dynamical systems for example molecular dynamics^44^.

#### Example 1

The Langevin equation

8 $$\begin{array}{ll}m\ddot{x}&=-\nabla U(x)-m{\gamma }_{1}\dot{x}+\sqrt{2m{\gamma }_{1}{k}_{{\mathrm{B}}}T}R(t),\\ \end{array}$$

can be written in the form of equation (6), where the dot denotes a time derivative, $\dot{x}$ is the velocity, $\ddot{x}$ is the acceleration, *m* is the mass, *U*(*x*) is the particle interaction potential, and so −∇*U*(*x*) is the potential force; *γ*_1_ is the damping constant (units of reciprocal time), $R(t)=\dot{B}(t)$ is a delta-correlated stationary Gaussian process with zero-mean, satisfying

$$\left\langle R(t)\right\rangle =0,\quad \left\langle R(t)R({t}^{{\prime} })\right\rangle =\delta (t-{t}^{{\prime} }).$$

If we set $\dot{x}=v$, the Langevin equation can be written as

$$\begin{array}{r}\left(\begin{array}{cc}m{\gamma }_{1}&m\\ -m&0\\ \end{array}\right)\left(\begin{array}{c}{\mathrm{d}}x\\ {\mathrm{d}}v\\ \end{array}\right)=-\left(\begin{array}{c}\nabla U(x)\\ mv\\ \end{array}\right){\mathrm{d}}t+\left(\begin{array}{c}\sqrt{2m{\gamma }_{1}{k}_{{\mathrm{B}}}T}\\ 0\\ \end{array}\right){\mathrm{d}}B(t).\,\end{array}$$

Denoting $${\boldsymbol{X}}=\left(\begin{array}{c}x\\ v\\ \end{array}\right),\tilde{M}=\left(\begin{array}{cc}m{\gamma }_{1}&0\\ 0&0\\ \end{array}\right),\tilde{W}=\left(\begin{array}{cc}0&m\\ -m&0\\ \end{array}\right),{{\varSigma }}=\left(\begin{array}{c}\sqrt{2m{\gamma }_{1}{k}_{{\mathrm{B}}}T}\\ 0\\ \end{array}\right)$$ and $V(x,v)=U(x)+\frac{m}{2}{v}^{2}$, the Langevin equation can be written in the form of the GSOP as follows:

$$\begin{array}{r}\left(\tilde{M}+\tilde{W}\right){\mathrm{d}}{\boldsymbol{X}}=-\nabla V{\mathrm{d}}t+{{\Sigma }}{\mathrm{d}}B.\end{array}$$

Another important class of dynamical systems are those described by Poisson brackets^45^, whose stochastic extension encompasses many applications, including the stochastic Lotka–Volterra models and variants^43^. In the following, we show that these dynamical systems can also be written in the form of the GSOP.

#### Example 2

The stochastic dynamics with generalized coordinates (*q*_1_, ... *q*_*n*_, *p*_1_, ... *p*_*n*_) described by generalized Poisson brackets

9 $$\begin{array}{r}{\mathrm{d}}F=(\{F,H\}-[F,H]){\mathrm{d}}t+\sigma (F){\mathrm{d}}B,\end{array}$$

can be written in the form of equation (7), where *H*(*q*_1_, …, *q*_*n*_; *p*_1_, …, *p*_*n*_) is the Hamiltonian of the system and *F* is an arbitrary function depending on the system variables. The Poisson bracket {⋅, ⋅} and the dissipation bracket [⋅, ⋅] are defined as

$$\begin{array}{rcl} \{F,H\}&=&\sum\limits_{i=1}^n\left(\frac{\partial F}{\partial q_i}\frac{\partial H}{\partial p_i}-\frac{\partial F}{\partial p_i}\frac{\partial H}{\partial q_i}\right)\\ {[}F, H{]} &=&J_F M J_H^T, \quad J_F=\left[\frac{\partial F}{\partial q_1},\cdots, \frac{\partial F}{\partial q_n},\frac{\partial F}{\partial p_1},\cdots,\frac{\partial F}{\partial p_n}\right], \end{array}$$

where *M* is symmetric positive semi-definite.

Denote (*h*_1_, *h*_2_,  …, *h*_2*n*_) = (*q*_1_,  …, *q*_*n*_, *p*_1_,  …, *p*_*n*_). By the definition of {*F*, *H*} and taking $$W=\left(\begin{array}{cc}0&-{I}_{n}\\ {I}_{n}&0\end{array}\right)$$, where *I*_n_ is the *n*-dimensional identity matrix, we have

$$\begin{array}{rc}\{F,H\}&=\left(\frac{\partial F}{\partial {\boldsymbol{q}}},\frac{\partial F}{\partial {\boldsymbol{p}}}\right)\left(\begin{array}{cc}0&{I}_{n}\\ -{I}_{n}&0\\ \end{array}\right)\left(\begin{array}{c}\frac{\partial H}{\partial {\boldsymbol{q}}}\\ \frac{\partial H}{\partial {\boldsymbol{p}}}\\ \end{array}\right)={\nabla }_{{\boldsymbol{h}}}F\left(\begin{array}{cc}0&{I}_{n}\\ -{I}_{n}&0\\ \end{array}\right){({\nabla }_{{\boldsymbol{h}}}H)}^{T}=-{{\boldsymbol{J}}}_{F}W{{\boldsymbol{J}}}_{H}^{T}.\end{array}$$

Hence, equation (9) can be written as

$$\begin{array}{r}{\mathrm{d}}F={({\nabla }_{{\boldsymbol{h}}}F\,)}^{T}{\mathrm{d}}{\boldsymbol{h}}=-{{\boldsymbol{J}}}_{F}(W+M\,){\,{\boldsymbol{J}}}_{H}^{T}{\mathrm{d}}t+\sigma (F\,){\mathrm{d}}B.\end{array}$$

Taking ***F*** = (*h*_1_, ..., *h*_2*n*_) and ∇_*h*_***F*** = *I*_2*n*_, we obtain

$$\begin{array}{r}{\mathrm{d}}{\boldsymbol{h}}=-{{\boldsymbol{J}}}_{F}(W+M\,){\,{\boldsymbol{J}}}_{H}^{T}{\mathrm{d}}t+\sigma (F\,){\mathrm{d}}B=-(W+M\,){\nabla }_{{\boldsymbol{h}}}H{\mathrm{d}}t+\sigma ({\boldsymbol{h}}){\mathrm{d}}B.\end{array}$$

Next, let us consider the reduction of a microscopic dynamical system satisfying a GSOP (***X***(*t*)) into a macroscopic dynamical system (***Z***(*t*)). This is achieved by a differentiable reduction function $\phi :{{\mathbb{R}}}^{D}\to {{\mathbb{R}}}^{d}$ such that ***Z***(*t*) ≈ $\varphi$ (***X***(*t*)). Moreover, we consider a differentiable reconstruction function $\psi :{{\mathbb{R}}}^{d}\to {{\mathbb{R}}}^{D}$ such that ***X***(*t*) ≈ *ψ*(***Z***(*t*)). Our main result is that *Z*(*t*) also satisfies an approximate GSOP. In other words, the GSOP family is approximately invariant under dimensionality reduction, or coordinate transformation in general.

In the following, we adopt the notation

$$\begin{array}{rcl}\nabla {\phi }_{i}({\boldsymbol{X}}(t))&:= &{\left(\frac{\partial {\phi }_{i}({\boldsymbol{X}}(t))}{\partial {x}_{1}},\frac{\partial {\phi }_{i}({\boldsymbol{X}}(t))}{\partial {x}_{2}},\cdots ,\frac{\partial {\phi }_{i}({\boldsymbol{X}}(t))}{\partial {x}_{D}}\right)}^{T},\\ \nabla \phi ({\boldsymbol{X}}(t))&:= &\left(\nabla {\phi }_{1}({\boldsymbol{X}}(t)),\cdots \,,\nabla {\phi }_{d}({\boldsymbol{X}}(t))\right).\end{array}$$

We will also adopt the following technical assumptions.

#### Assumption 1

The functions ${M}_{1},{W}_{1},\nabla {V}_{1}:{{\mathbb{R}}}^{D}\to {{\mathbb{R}}}^{D}$, ${{{\varSigma }}}_{1}:{{\mathbb{R}}}^{D}\to {{\mathbb{R}}}^{D\times {p}_{1}}$, $\phi :{{\mathbb{R}}}^{D}\to {{\mathbb{R}}}^{d}$ and $\psi :{{\mathbb{R}}}^{d}\to {{\mathbb{R}}}^{D}$ satisfy:

1. Growth condition: there exists a positive constasnt *L* > 0 such that, for all ${\boldsymbol{x}}\in {{\mathbb{R}}}^{D}$ and ${\boldsymbol{z}}\in {{\mathbb{R}}}^{d}$
  $$\begin{array}{rcl}&&| ({M}_{1}({\boldsymbol{x}})+{W}_{1}({\boldsymbol{x}}))\nabla {V}_{1}({\boldsymbol{x}})| +| {{{\varSigma }}}_{1}({\boldsymbol{x}})| +\mathop{\sum }\limits_{i=1}^{d}| \nabla {\phi }_{i}({\boldsymbol{x}})| \le {L}^{2}(1+| {\boldsymbol{x}}| ),\\ &&\mathop{\sum }\limits_{i=1}^{D}| \nabla {\psi }_{i}({\boldsymbol{z}})| +| \psi ({\boldsymbol{z}})| \le {L}^{2}(1+| {\boldsymbol{z}}| ),\,\end{array}$$
2. Lipschitz condition: there exists a positive constant *L* > 0 such that, for all ${\boldsymbol{x}}\in {{\mathbb{R}}}^{D}$, and ${\boldsymbol{z}}\in {{\mathbb{R}}}^{d}$, the function $({M}_{1}({\boldsymbol{x}})+{W}_{1}({{x}}))\nabla {V}_{1}({\boldsymbol{x}}),{{{\varSigma }}}_{1}({\boldsymbol{x}}),$ ${\{\nabla {\psi }_{i}(z)\}}_{i = 1}^{D}$ and *ψ*(*z*) satisfy the Lipschitz condition with constant *L*.
3. Approximate reconstruction: there exists $\varepsilon_0$ > 0 such that, $\mathop{\sup }\limits_{{\boldsymbol{x}}\in {{\varOmega }}}| {\boldsymbol{x}}-\psi (\phi ({\boldsymbol{x}}))| < {\epsilon }_{0}$ where ${{\varOmega }}\subset {{\mathbb{R}}}^{D}$ is a domain such that ***X***(*t*) ∈ *Ω* for all *t* ∈ [0, *T*] almost surely.

Here, |⋅| is the Euclidean norm for a vector and Frobenius norm for a matrix.

#### Theorem 1

Let ***X***(*t*) satisfy equation (7) and ***Z***(*t*) satisfy equation (5) with

$$\begin{array}{rcl}M({\boldsymbol{Z}}\,)&=&\nabla \phi {[\psi ({\boldsymbol{Z}}\,)]}^{T}{M}_{1}(\psi ({\boldsymbol{Z}}\,))\nabla \phi [\psi ({\boldsymbol{Z}}\,)],\\ W({\boldsymbol{Z}}\,)&=&\nabla \phi {[\psi ({\boldsymbol{Z}}\,)]}^{T}{W}_{1}(\psi ({\boldsymbol{Z}}\,))\nabla \phi [\psi ({\boldsymbol{Z}}\,)],\\ V({\boldsymbol{Z}}\,)&=&{V}_{1}[\psi ({\boldsymbol{Z}}\,)],\\ {\sigma }_{1}({\boldsymbol{Z}}\,)&=&\sqrt{{\epsilon }_{1}}\nabla \phi {[\psi ({\boldsymbol{Z}}\,)]}^{T}{{{\varSigma }}}_{1}[\psi ({\boldsymbol{Z}}\,)],\\ \sigma ({\boldsymbol{Z}}\,)&=&{[{\sigma }_{1}({\boldsymbol{Z}}\,){\sigma }_{1}{({\boldsymbol{Z}}\,)}^{T}]}^{\frac{1}{2}},\end{array}$$

Then, for each $u\in {{{{\mathcal{C}}}}}^{2}({{\mathbb{R}}}^{d})$ (twice continuously differentiable function) there exists a constant *C* > 0, independent of $\varepsilon_0$ and $\varepsilon_1$, such that

$$\begin{array}{r}| {\mathbb{E}}u(\phi [{\boldsymbol{X}}(t)])-{\mathbb{E}}u({\boldsymbol{Z}}(t))| \le C({\epsilon }_{0}+{\epsilon }_{1}).\,\end{array}$$

#### Proof

Suppose ***Y***(*t*) satisfy the following equation:

10 $$\begin{array}{rc}{\mathrm{d}}{\boldsymbol{Y}}(t)&=-\left.\right(M({\boldsymbol{Y}}(t)+W({\boldsymbol{Y}}(t)))\nabla V({\boldsymbol{Y}}(t)){\mathrm{d}}t+{\sigma }_{1}({\boldsymbol{Y}}(t)){\mathrm{d}}{{\boldsymbol{B}}}_{1}(t),\end{array}$$

we will prove $$\begin{array}{r}{\mathbb{E}}| u(\phi [{\boldsymbol{X}}(t)])-u({\boldsymbol{Y}}(t))| \le C({\epsilon }_{0}+{\epsilon }_{1}).\,\end{array}$$By Itô’s formula, equation (7) and Assumption 1, we obtain

11 $$\begin{array}{rcl}{\mathrm{d}}{\phi }_{i}({\boldsymbol{X}}(t))&=&{[\nabla {\phi }_{i}({\boldsymbol{X}}(t))]}^{T}{\mathrm{d}}{\boldsymbol{X}}(t)+\frac{1}{2}{[{\mathrm{d}}{\boldsymbol{X}}(t)]}^{T}{\nabla }^{2}{\phi }_{i}({\boldsymbol{X}}(t)){\mathrm{d}}{\boldsymbol{X}}(t)\\ &=&{[\nabla {\phi }_{i}({\boldsymbol{X}}(t))]}^{T}\left[-\left({M}_{1}({\boldsymbol{X}}(t))+{W}_{1}({\boldsymbol{X}}(t))\right)\nabla {V}_{1}({\boldsymbol{X}}(t)){\mathrm{d}}t\right.\\&&\left.+\sqrt{{\epsilon }_{1}}{{{\varSigma }}}_{1}({\boldsymbol{X}}(t)){\mathrm{d}}{B}_{1}(t)\right]\\ &&+\frac{{\epsilon }_{1}}{2}{{{\rm{Tr}}}}[{{{\varSigma }}}_{1}({\boldsymbol{X}}(t)){{{\varSigma }}}_{1}^{T}({\boldsymbol{X}}(t)){\nabla }^{2}{\phi }_{i}({\boldsymbol{X}}(t))]{\mathrm{d}}t,\end{array}$$

where ∇^2^$\phi_i$ is the Hessian matrix of $\phi_i$ and Tr is the trace of a square matrix.

Now, by definition, ***Y***(*t*) satisfies the following stochastic differential equation (SDE)

12 $$\begin{array}{rcl}{\mathrm{d}}{\boldsymbol{Y}}(t)&=&-\left(M({\boldsymbol{Y}}(t))+W({\boldsymbol{Y}}(t))\right)\nabla V({\boldsymbol{Y}}(t)){\mathrm{d}}t+{\sigma }_{1}({\boldsymbol{Y}}(t)){\mathrm{d}}{B}_{1}(t)\\ &=&-\nabla \phi {[\psi ({\boldsymbol{Y}}(t))]}^{T}[{M}_{1}(\psi ({\boldsymbol{Y}}(t)))+{W}_{1}(\psi ({\boldsymbol{Y}}(t)))]\nabla \phi [\psi ({\boldsymbol{Y}}(t))]\nabla V({\boldsymbol{Y}}(t)){\mathrm{d}}t\\ &&+\sqrt{{\epsilon }_{1}}\nabla \phi {[\psi ({\boldsymbol{Y}}(t))]}^{T}{{{\varSigma }}}_{1}(\psi ({\boldsymbol{Y}}(t))){\mathrm{d}}{B}_{1}(t)\\ &=&-\nabla \phi {[\psi ({\boldsymbol{Y}}(t))]}^{T}[{M}_{1}(\psi ({\boldsymbol{Y}}(t)))+{W}_{1}(\psi ({\boldsymbol{Y}}(t)))]\nabla {V}_{1}(\psi ({\boldsymbol{Y}}(t))){\mathrm{d}}t\\ &&+\sqrt{{\epsilon }_{1}}\nabla \phi {[\psi ({\boldsymbol{Y}}(t))]}^{T}{{{\varSigma }}}_{1}(\psi ({\boldsymbol{Y}}(t))){\mathrm{d}}{B}_{1}(t).\,\end{array}$$

Subtracting equations (11) and (12), and integrating on [0, *t*], we get

$$\begin{array}{l}{\phi }_{i}({\boldsymbol{X}}(t))-{Y}_{i}(t)\\=\displaystyle\int\nolimits_{0}^{t}\left[\nabla {\phi }_{i}{({\boldsymbol{X}}(r))}^{T}{f}_{1}({\boldsymbol{X}}(r))-\nabla {\phi }_{i}{[\psi ({\boldsymbol{Y}}(r))]}^{T}{f}_{1}(\psi ({\boldsymbol{Y}}(r)))\right]{\mathrm{d}}r\\+{\epsilon }_{1}\displaystyle\int\nolimits_{0}^{t}{g}_{i}({\boldsymbol{X}}(r)){\mathrm{d}}r+\sqrt{{\epsilon }_{1}}\displaystyle\int\nolimits_{0}^{t}\left[\nabla {\phi }_{i}{({\boldsymbol{X}}(r))}^{T}{{{\varSigma }}}_{1}({\boldsymbol{X}}(r))\right.\\\left.-\nabla {\phi }_{i}{[\psi ({\boldsymbol{Y}}(r))]}^{T}{{{\varSigma }}}_{1}(\psi ({\boldsymbol{Y}}(r)))\right]{\mathrm{d}}{{\boldsymbol{B}}}_{1}(r),\end{array}$$

where ${f}_{1}(\cdot )=-\left({M}_{1}(\cdot )+{W}_{1}(\cdot )\right)\nabla {V}_{1}(\cdot )$ and ${g}_{i}(\cdot )=\frac{1}{2}{{{\rm{Tr}}}}[{{{\varSigma }}}_{1}{{{\varSigma }}}_{1}^{T}{\nabla }^{2}{\phi }_{i}](\cdot )$ with *i* = 1, 2, ..., *d*.

By Cauchy–Schwarz inequality and Itô isometry, we have

13 $$\begin{array}{l}{\mathbb{E}}| {\phi }_{i}({\boldsymbol{X}}(t))-{Y}_{i}(t){| }^{2}\\ \le 3{\mathbb{E}}{\left\vert \displaystyle\int\nolimits_{0}^{t}[\nabla {\phi }_{i}{({\boldsymbol{X}}(r))}^{T}{f}_{1}({\boldsymbol{X}}(r))-\nabla {\phi }_{i}{[\psi ({\boldsymbol{Y}}(r))]}^{T}{f}_{1}(\psi ({\boldsymbol{Y}}(r)))]{\mathrm{d}}r\right\vert }^{2}\\+3{\mathbb{E}}{\left\vert \displaystyle\int\nolimits_{0}^{t}{\epsilon }_{1}{g}_{i}({\boldsymbol{X}}(r)){\mathrm{d}}r\right\vert }^{2}\\+3{\epsilon }_{1}{\mathbb{E}}{\left\vert \displaystyle\int\nolimits_{0}^{t}[\nabla {\phi }_{i}({\boldsymbol{X}}(r)){{{\varSigma }}}_{1}({\boldsymbol{X}}(r))-\nabla {\phi }_{i}{[\psi ({\boldsymbol{Y}}(r))]}^{T}{{{\varSigma }}}_{1}(\psi ({\boldsymbol{Y}}(r)))]{\mathrm{d}}{B}_{1}(r)\right\vert }^{2}\\ \le 3t{\mathbb{E}}\displaystyle\int\nolimits_{0}^{t}{\left\vert \nabla {\phi }_{i}{({\boldsymbol{X}}(r))}^{T}{f}_{1}({\boldsymbol{X}}(r))-\nabla {\phi }_{i}{[\psi ({\boldsymbol{Y}}(r))]}^{T}{f}_{1}(\psi ({\boldsymbol{Y}}(r)))\right\vert }^{2}{\mathrm{d}}r\\+3{\epsilon }_{1}^{2}t{\mathbb{E}}\displaystyle\int\nolimits_{0}^{t}| {g}_{i}({\boldsymbol{X}}(r)){| }^{2}{\mathrm{d}}r\\ +3{\epsilon }_{1}{\mathbb{E}}\displaystyle\int\nolimits_{0}^{t}{\left\vert \nabla {\phi }_{i}({\boldsymbol{X}}(r)){{{\varSigma }}}_{1}({\boldsymbol{X}}(r))-\nabla {\phi }_{i}{[\psi ({\boldsymbol{Y}}(r))]}^{T}{{{\varSigma }}}_{1}(\psi ({\boldsymbol{Y}}(r)))\right\vert }^{2}{\mathrm{d}}r.\,\end{array}$$

By Assumption 1, there exists positive constants *C*_1_ and *C*_2_ such that

$$\begin{array}{l}{\mathbb{E}}\displaystyle\int\nolimits_{0}^{t}| \nabla {\phi }_{i}{({\boldsymbol{X}}(r))}^{T}{f}_{1}({\boldsymbol{X}}(r))-\nabla {\phi }_{i}{[\psi ({\boldsymbol{Y}}(r))]}^{T}{f}_{1}(\psi ({\boldsymbol{Y}}(r))){| }^{2}{\mathrm{d}}r\\ \le {\mathbb{E}}\displaystyle\int\nolimits_{0}^{t}| \nabla {\phi }_{i}{({\boldsymbol{X}}(r))}^{T}{f}_{1}({\boldsymbol{X}}(r))-\nabla {\phi }_{i}{[\psi (\phi ({\boldsymbol{X}}(r)))]}^{T}{f}_{1}({\boldsymbol{X}}(r))\\+\nabla {\phi }_{i}{[\psi (\phi ({\boldsymbol{X}}(r)))]}^{T}{f}_{1}({\boldsymbol{X}}(r))\\ -\nabla {\phi }_{i}{[\psi ({\boldsymbol{Y}}(r))]}^{T}{f}_{1}({\boldsymbol{X}}(r))+\nabla {\phi }_{i}{[\psi ({\boldsymbol{Y}}(r))]}^{T}{f}_{1}({\boldsymbol{X}}(r))\\-\nabla {\phi }_{i}{[\psi ({\boldsymbol{Y}}(r))]}^{T}{f}_{1}(\psi (\phi ({\boldsymbol{X}}(r))))\\+\nabla {\phi }_{i}{[\psi ({\boldsymbol{Y}}(r))]}^{T}{f}_{1}(\psi (\phi ({\boldsymbol{X}}(r))))-\nabla {\phi }_{i}{[\psi ({\boldsymbol{Y}}(r))]}^{T}{f}_{1}(\psi ({\boldsymbol{Y}}(r))){| }^{2}{\mathrm{d}}r\\ \le {C}_{1}{\mathbb{E}}\displaystyle\int\nolimits_{0}^{t}| {\boldsymbol{X}}(r)-\psi (\phi ({\boldsymbol{X}}(r))){| }^{2}{\mathrm{d}}r+{C}_{2}{\mathbb{E}}\displaystyle\int\nolimits_{0}^{t}| \phi ({\boldsymbol{X}}(r))-{\boldsymbol{Y}}(r){| }^{2}{\mathrm{d}}r\\ \le {C}_{1}t{\epsilon }_{0}^{2}+{C}_{2}{\mathbb{E}}\displaystyle\int\nolimits_{0}^{t}| \phi ({\boldsymbol{X}}(r))-{\boldsymbol{Y}}(r){| }^{2}{\mathrm{d}}r.\,\end{array}$$

We employ a similar above argument of the third term of equation (13) and get

$$\begin{array}{rc}{\mathbb{E}}| \phi ({\boldsymbol{X}}(t))-{\boldsymbol{Y}}(t){| }^{2}\le &{C}_{3}\displaystyle\int\nolimits_{0}^{t}{\mathbb{E}}| \phi ({\boldsymbol{X}}(r))-{\boldsymbol{Y}}(r){| }^{2}{\mathrm{d}}r+{C}_{4}{({\epsilon }_{0}+{\epsilon }_{1})}^{2}.\,\end{array}$$

Here we have used the Lipschitz conditions and the boundness of the first moment of *f*_1_(*X*) and *g*_*i*_(*X*), which is implied by the growth condition in Assumption 1. This shows that $\phi$ (*X*(*t*)) and *Y*(*t*) are close in the mean-square sense. By Gronwall’s inequality, we get

14 $$\begin{array}{rc}{\mathbb{E}}| \phi ({\boldsymbol{X}}(t))-{\boldsymbol{Y}}(t){| }^{2}\le &{C}_{5}{({\epsilon }_{0}+{\epsilon }_{1})}^{2}.\,\end{array}$$

Now, we employ a similar argument to show that *u*($\phi$(***X***(*t*))) and *u*(***Y*(***t*)) are close for any sufficiently smooth *u*. We apply Itô formula to *u*(*φ*(***X***(*t*))) and *u*(***Y*(***t*)) to get

$$\begin{array}{l}{\mathrm{d}}u(\phi ({\boldsymbol{X}}(t)))=\nabla u\cdot {\mathrm{d}}\phi ({\boldsymbol{X}}(t))+\frac{1}{2}{\mathrm{d}}\phi {({\boldsymbol{X}}(t))}^{T}{\nabla }^{2}u\left(\phi ({\boldsymbol{X}}(t)){\mathrm{d}}\phi ({\boldsymbol{X}}(t))\right.\\\qquad\qquad\quad\,\,\,=\nabla u\cdot \left[\right.(\nabla \phi {({\boldsymbol{X}}(t))}^{T}{f}_{1}({\boldsymbol{X}}(t))+{\epsilon }_{1}g({\boldsymbol{X}}(t))){\mathrm{d}}t\\\qquad\qquad\quad\,\,\,+\left.\nabla \phi ({\boldsymbol{X}}(t)){{{\varSigma }}}_{1}({\boldsymbol{X}}(t)){\mathrm{d}}{B}_{1}(t)\right]\\\qquad\qquad\quad\,\,\,+\frac{1}{2}{\epsilon }_{1}{{{\rm{Tr}}}}[\nabla \phi ({\boldsymbol{X}}(t)){{{\varSigma }}}_{1}({\boldsymbol{X}}(t))(\nabla \phi ({\boldsymbol{X}}(t)){{{\varSigma }}}_{1}{({\boldsymbol{X}}(t))}^{T}{\nabla }^{2}u]){\mathrm{d}}t,\\\qquad\qquad\quad\,\,\,{\mathrm{d}}u({\boldsymbol{Y}}(t))=\nabla u\cdot {\mathrm{d}}{\boldsymbol{Y}}(t)+\frac{1}{2}{\mathrm{d}}{\boldsymbol{Y}}{(t)}^{T}{\nabla }^{2}u({\boldsymbol{Y}}(t)){\mathrm{d}}{\boldsymbol{Y}}(t)\\\qquad\qquad\quad\,\,\, =\nabla u\cdot \nabla \phi {[\psi ({\boldsymbol{Y}}(t))]}^{T}[{f}_{1}(\psi ({\boldsymbol{Y}}(t))){\mathrm{d}}t+\sqrt{{\epsilon }_{1}}{{{\varSigma }}}_{1}(\psi ({\boldsymbol{Y}}(t))){\mathrm{d}}{B}_{1}(t)]\\\qquad\qquad\quad\,\,\, +\frac{{\epsilon }_{1}}{2}{{{\rm{Tr}}}}[\nabla \phi [\psi ({\boldsymbol{Y}}(t))]{{{\varSigma }}}_{1}(\psi ({\boldsymbol{Y}}(t))){(\nabla \phi [\psi ({\boldsymbol{Y}}(t))]{{{\varSigma }}}_{1}(\psi ({\boldsymbol{Y}}(t))))}^{T}{\nabla }^{2}u]{\mathrm{d}}t.\, \end{array}$$

As before, by Assumption 1, there exist positive constants *C*_6_ and *C*_7_ such that

15 $$\begin{array}{rc}{\mathbb{E}}| u(\phi ({\boldsymbol{X}}(t)))-u({\boldsymbol{Y}}(t)){| }^{2}\le &{C}_{6}{\mathbb{E}}\displaystyle\int_{0}^{t}| \left.\phi ({\boldsymbol{X}}(r))\right)-{\boldsymbol{Y}}(r){| }^{2}{\mathrm{d}}r+{C}_{7}{({\epsilon }_{0}+{\epsilon }_{1})}^{2}.\end{array}$$

Combining equations (14) and (15), we obtain

$$\begin{array}{r}{\mathbb{E}}| u(\phi ({\boldsymbol{X}}(t)))-u({\boldsymbol{Y}}(t)){| }^{2}\le {C}^{2}{({\epsilon }_{0}+{\epsilon }_{1})}^{2}.\,\end{array}$$

By Jensen’s inequality, we can get

$$\begin{array}{r}{\mathbb{E}}| u(\phi ({\boldsymbol{X}}(t)))-u({\boldsymbol{Y}}(t))| \le C({\epsilon }_{0}+{\epsilon }_{1}).\,\end{array}$$

According to the equations (5) and (10), we can get *Z*(*t*) and *Y*(*t*) has the some distribution, that is

$$\begin{array}{r}{\mathbb{E}}u({\boldsymbol{Y}}(t))-{\mathbb{E}}u({\boldsymbol{Z}}(t))=0.\,\end{array}$$

Finally, by triangle inequality, we have

$$\begin{array}{rcl}&&| {\mathbb{E}}u(\phi ({\boldsymbol{X}}(t)))-{\mathbb{E}}u({\boldsymbol{Y}}(t))| \\ &=&| {\mathbb{E}}u(\phi ({\boldsymbol{X}}(t)))-{\mathbb{E}}u({\boldsymbol{Y}}(t))+{\mathbb{E}}u({\boldsymbol{Y}}(t))-{\mathbb{E}}u(Z(t))| \\ &\le &{\mathbb{E}}| u(\phi ({\boldsymbol{X}}(t)))-u({\boldsymbol{Y}}(t))| +| {\mathbb{E}}u({\boldsymbol{Y}}(t))-{\mathbb{E}}u(Z(t))| \\ &\le &C({\epsilon }_{0}+{\epsilon }_{1}).\,\end{array}$$

This completes the proof.

This results demonstrate the validity of the GSOP as a dimensionality reduction method. In short, it says that if the microscopic dynamics satisfies a GSOP, then the macroscopic dynamics will also satisfy a GSOP approximately. As a large amount of conservative and dissipative microscopic physical systems are shown to satisfy the GSOP, the S-OnsagerNet approach based on the GSOP is a principled model reduction ansatz for physical processes.

Next, we show the stability of a solution of the GSOP. More precisely, we prove in Theorem 2 below that the mean of the potential is non-increasing in *t* for sufficiently low temperatures (small |*σ*|). Consequently, the S-OnsagerNet produces dissipative dynamical systems that enjoy long-term stability.

#### Theorem 2

The solution of equation (5) satisfies the dissipation law

$$\begin{array}{rcl}{\mathbb{E}}V({\boldsymbol{Z}}(t))-{\mathbb{E}}V({\boldsymbol{Z}}(0))&=&-\displaystyle\int\nolimits_{0}^{t}{\mathbb{E}}\parallel \nabla V({\boldsymbol{Z}}(r)){\parallel }_{M}^{2}{\mathrm{d}}r\\ &&+\frac{1}{2}\displaystyle\int\nolimits_{0}^{t}{\mathbb{E}}{{{\rm{Tr}}}}[\sigma ({\boldsymbol{Z}}(r))\sigma {({\boldsymbol{Z}}(r))}^{T}{\nabla }^{2}V({\boldsymbol{Z}}(r))]{\mathrm{d}}r.\,\end{array}$$

Here $\parallel \cdot {\parallel }_{M}^{2}$ denotes |*M*^1/2^⋅|^2^ where *M*^1/2^ is the non-negative square-root of *M*. If we assume further that there exists a positive constant *α* such that ***Z***^T^ *M*(***Z***)***Z*** ≥ *α*|*Z*|^2^ and $\frac{1}{2}{{{\rm{Tr}}}}[\sigma (Z\,)\sigma {(Z\,)}^{T}{\nabla }^{2}V(Z\,)]\le \alpha | \nabla V(Z\,){| }^{2}$ for all *Z*, then ${\mathbb{E}}[V(Z(t))]$ is non-increasing in *t*.

#### Proof

By Itô formula, we obtain

16 $$\begin{array}{rcl}{\mathrm{d}}V({\boldsymbol{Z}}\,)&=&\nabla V\cdot {\mathrm{d}}{\boldsymbol{Z}}+\frac{1}{2}{({\mathrm{d}}{\boldsymbol{Z}})}^{T}{\nabla }^{2}V{\mathrm{d}}{\boldsymbol{Z}}\\ &=&-\nabla V\cdot [(M+W)\nabla V]{\mathrm{d}}t+\nabla V\cdot \sigma {\mathrm{d}}{{\boldsymbol{B}}}_{1}+\frac{1}{2}{{{\rm{Tr}}}}[\sigma {\sigma }^{T}{\nabla }^{2}V]{\mathrm{d}}t\\ &=&-\parallel \nabla V{\parallel }_{M}^{2}{\mathrm{d}}t+\frac{1}{2}{{{\rm{Tr}}}}[\sigma {\sigma }^{T}{\nabla }^{2}V]{\mathrm{d}}t+\nabla V\cdot \sigma {\mathrm{d}}{{\boldsymbol{B}}}_{1},\end{array}$$

where $\nabla V^T M\nabla V=\parallel \nabla V{\parallel }_{M}^{2}$ and ∇*V*^T^ *W*∇*V* = 0 are used.

Integrating equation (16) from 0 to *t* and taking expectation, we obtain

$$\begin{array}{rc}{\mathbb{E}}V({\boldsymbol{Z}}(t))-{\mathbb{E}}V({\boldsymbol{Z}}(0))&=-\displaystyle\int\nolimits_{0}^{t}{\mathbb{E}}\parallel \nabla V{\parallel }_{M}^{2}{\mathrm{d}}r+\frac{1}{2}\int\nolimits_{0}^{t}{\mathbb{E}}{{{\rm{Tr}}}}\left[\sigma {\sigma }^{T}{\nabla }^{2}V\right]{\mathrm{d}}r.\,\end{array}$$

Finally, according to the proposed condition, it is easy to arrive at $$\begin{array}{rc}{\mathbb{E}}V({\boldsymbol{Z}}(t))-{\mathbb{E}}V({\boldsymbol{Z}}(0))&\le -\alpha \displaystyle\int\nolimits_{0}^{t}{\mathbb{E}}| \nabla V{| }^{2}{\mathrm{d}}r+\frac{1}{2}\int\nolimits_{0}^{t}{\mathbb{E}}{{{\rm{Tr}}}}[\sigma {\sigma }^{T}{\nabla }^{2}V]{\mathrm{d}}r\le 0,\end{array}$$thus the mean of the energy potential is non-increasing in *t*. This completes the proof.

Theorem 2 shows that energy is being dissipated if the temperature of the ambient reservoir is sufficiently low. Accordingly, if the free energy *V* has compact level sets, then the dynamics at low temperatures will be confined on average to these compact sets and is thus stable. This contrasts with unstructured methods that may learn dynamics that are accurate for short times but induce instability at long times. We numerically verify this energy dissipation law in the learned polymer dynamics in Supplementary Fig. 1.

### Model implementation

We provide in this section the detailed implementation of S-OnsagerNet.

#### Model architecture

We begin with discussing the architecture design of the neural network approximators. Following the acquisition of the closure coordinates ${\boldsymbol{Z}}(t)=({\boldsymbol{Z}}^{* }(t),\hat{{\boldsymbol{Z}}}(t))$, the S-OnsagerNet architecture implements equation (5). To ensure the symmetric positive definiteness of *M*(***Z***) and the anti-symmetry of *W*(***Z***), we use a neural network to approximate $A(\cdot ):{{\mathbb{R}}}^{d}\to {{\mathbb{R}}}^{{d}^{2}}$ with dimension *d*^2^. Then, we take the lower-triangular part as *L*_1_(***Z***) and the upper-triangular part as *L*_2_(***Z***). *M*(***Z***) and *W*(***Z***) are represented by

$$\begin{array}{rcl}M({\boldsymbol{Z}}\,)&=&{L}_{1}({\boldsymbol{Z}}\,){L}_{1}{({\boldsymbol{Z}}\,)}^{T}+\alpha I,\\ W({\boldsymbol{Z}}\,)&=&{L}_{2}({\boldsymbol{Z}}\,)-{L}_{2}{({\boldsymbol{Z}}\,)}^{T},\end{array}$$

where *α* is a positive constant and *I* is an identity matrix.

The energy function *V*(⋅) is lower bounded, so we use the following structure

$$\begin{array}{r}V({\boldsymbol{Z}}\,)=\frac{1}{2}\mathop{\sum }\limits_{i=1}^{m}{\left({U}_{i}({\boldsymbol{Z}}\,)+\mathop{\sum }\limits_{j = 1}^{d}{\gamma }_{ij}{{\boldsymbol{Z}}}_{j}\right)}^{2}+\beta | {\boldsymbol{Z}}{| }^{2},\end{array}$$

where *U*(***Z***) is a neural network with *d*-dimensional input and *m*-dimensional output, {*γ*_*ij*_} is a trainable matrix and *β* is a positive parameter.

In the architecture used for the polymer dynamics application in this paper, we set *α* = 0.1 and utilize a neural network with 2 hidden layers with 20 neurons each and the tanh activation function to approximate *M*(***z***) and *W*(***z***). To parameterize the potential *V*(*z*), we decompose it into a sum of squares of the output layer (size *m* = 50) of 1 hidden layer neural network with 128 hidden neurons and the ReQUr activation function^7,46^. This is to ensure that the potential satisfies the correct growth conditions as outlined in Assumption 1.

For the diffusion matrix *σ*(***z***), as it has no symmetry constraints other than a growth condition, we use a fully connected neural network to approximate it. In our polymer dynamics application, we found empirically that a diagonal, *z*-independent diffusion matrix (corresponding to a linear network with zero weight and trainable diagonal bias) performed the best, but our algorithm can handle general architectures for *σ*(***z***).

#### Closure coordinate construction

We now provide details of the procedure to construct closure coordinates $$\hat{{\boldsymbol{Z}}}(t)$$ using the time series observation data of chain configuration coordinates at $${\{{t}_{k}\}}_{k = 1}^{{N}_{t}}$$, with $$0={t}_{0} < {t}_{1}\ldots < {t}_{{N}_{t}}=T$$. The available data are $${\{{{{{\mathcal{X}}}}}_{j}\}}_{j = 1}^{M}$$ with $${{{{\mathcal{X}}}}}_{j}=$$$${\{{\boldsymbol{X}}{({t}_{i})}^{(\,j)}\}}_{i = 1}^{{N}_{t}}\in {{\mathbb{M}}}^{D\times {N}_{t}}$$, where *M* is the number of trajectories and $${\boldsymbol{X}}{({t}_{i})}^{(\,j)}$$ is the *j*th observation trajectory at *t* = *t*_*i*_. We obtained 610 observational trajectories, and for each trajectory, the number of time snapshots is 1,001, that is *M* = 610 and *N*_*t*_ = 1,001. We reshape the observation data as $$\Xi=[{{{{\mathcal{X}}}}}_{1},{{{{\mathcal{X}}}}}_{2},\ldots ,{{{{\mathcal{X}}}}}_{M}]$$, where $$\Xi\in {{\mathbb{M}}}^{D\times {N}_{t}M}$$. We re-center the data, such that the mean of the training data is zero for each time snapshot, and set it as *X*. The covariance matrix of *X* is *S* = Cov(*X*). Denote its eigenvalues by *Λ* = diag(*λ*_1_, *λ*_2_, …, *λ*_*D*_) (arranged in non-increasing order) and corresponding eigenvectors as *V* = (***V***_1_, ***V***_2_, …, ***V***_*D*_).

We use the following PCA-ResNet encoder to find the closure coordinates

17 $$\begin{array}{r}\hat{{\boldsymbol{Z}}}(t)={{\boldsymbol{Z}}}_{2:d}(t)=\hat{\phi }({\boldsymbol{X}}(t))={P}_{d}{\boldsymbol{X}}(t)+{{{{\rm{NN}}}}}_{e}({\boldsymbol{X}}(t)),\end{array}$$

where ${P}_{d}=\frac{1}{\sqrt{{{{\varLambda }}}_{1:d-1}}}{V}_{1:d-1}$ and NN_*e*_(⋅) is a fully connected neural network with input dimension *D* and output dimension *d* − 1. We can reconstruct the high-dimensional coordinates via the decoder

18 $$\begin{array}{r}\tilde{{\boldsymbol{X}}}(t)=\psi (\hat{{\boldsymbol{Z}}}(t))={P}_{d}^{{\dagger} }\hat{{\boldsymbol{Z}}}(t)+{{{{\rm{NN}}}}}_{d}(\hat{{\boldsymbol{Z}}}(t)),\end{array}$$

where ${P}_{d}^{{\dagger} }={V}_{1:d-1}^{T}\sqrt{{{{\varLambda }}}_{1:d-1}}$ and ${{{{\rm{NN}}}}}_{d}(\hat{{\boldsymbol{Z}}}(t))$ is another neural network with input dimension *d* − 1 and output dimension *D*. Note that without NN_*e*_, NN_*d*_, this amounts to a PCA-based coordinate reduction. The combination of PCA and neural networks combines approximate feature orthogonality and approximation flexibility.

We construct the reconstruction loss function as $| {\boldsymbol{X}}-\tilde{{\boldsymbol{X}}}{| }^{2}$. We set the reconstruction error of PCA as ${E}_{{\mathrm{pca}}}=| {\boldsymbol{X}}-{P}_{d}^{{\dagger} }{P}_{d}{\boldsymbol{X}}{| }^{2}$. To make the reconstruction error near but less than the reconstruction error of PCA alone, we add the regularization term ${{{\rm{ReLU}}}}(\log | {\boldsymbol{X}}-\tilde{{\boldsymbol{X}}}{| }^{2}-\log ({E}_{{\mathrm{pca}}}))$ in the loss function, where ${E}_{{\mathrm{pca}}}=| {\boldsymbol{X}}-{P}_{d}^{{\dagger} }{P}_{d}{\boldsymbol{X}}{| }^{2}$ is the reconstruction error of PCA and ReLU is the rectified linear unit, that is ${{{\rm{ReLU}}}}(u)=\max (0,u)$. Thus, the combined reconstruction loss function is

$$\begin{array}{r}{{{{\rm{loss}}}}}_{{{{\rm{Rec}}}}}=| {\boldsymbol{X}}-\tilde{{\boldsymbol{X}}}{| }^{2}+{\rho }_{1}{{{\rm{ReLU}}}}(\log | {\boldsymbol{X}}-\tilde{{\boldsymbol{X}}}{| }^{2}-\log | {\boldsymbol{X}}-{P}_{d}^{{\dagger} }{P}_{d}{\boldsymbol{X}}{| }^{2}),\,\end{array}$$

where *ρ*_1_ is a regularization parameter. The regularization term penalizes the loss if we observe a reconstruction error that is higher than PCA.

#### Training algorithm based on maximum likelihood estimation

After constructing the structure of the drift term $$f({\boldsymbol{Z}}(t))=$$$$-\left.\right(M\left({\boldsymbol{Z}}(t)\right.$$ $$+W({\boldsymbol{Z}}(t))\left)\right.\nabla V({\boldsymbol{Z}}(t))$$ and diffusion term *σ*(***Z***(*t*)), we consider how to construct the loss function to learn the stochastic dynamics. In deterministic dynamical systems, we can use the mean square error to learn *f* given the trajectory observation data. However, to deal with stochastic dynamics (in particular, learning the diffusion matrix *σ*), we have to devise more general methods based on maximum likelihood estimation.

We discretize equation (5) by the Euler–Maruyama scheme, giving

$$\begin{array}{rc}{\boldsymbol{Z}}({t}_{i+1})&={\boldsymbol{Z}}({t}_{i})+f({\boldsymbol{Z}}({t}_{i})){{\Delta }}t+\sigma ({\boldsymbol{Z}}({t}_{i}))\sqrt{{{\Delta }}t}{\xi }_{i},\end{array}$$

where Δ*t* is the time step, *t*_*i*_ = *i*Δ*t*, *i* = 0, 1, …, *N*_*t*_ − 1 and *T* = *N*_*t*_Δ*t*. Here, ${\{{\boldsymbol{\xi} }_{i}\}}_{i = 0}^{{N}_{t}-1}$ are independent random vectors following the standard normal distribution.

In the training dataset, we have access to the microscopic coordinates ***X***(*t*), from which we construct the reduced coordinates ${\{(Z{({t}_{i})}^{(\;j)},Z({t}_{i+1}^{(\;j)}),{{\Delta }}t)\}}_{i,\,j = 0,1}^{{N}_{t}-1,M}$ via the (to be trained) reduction function $\phi$. Given the neural networks *f*_*θ*_ and *σ*_*θ*_ (we use the subscript *θ* to denote all trainable parameters) constructed previously, the conditional probability is given by

$$\begin{array}{rcl}&&p\left({\boldsymbol{Z}}{({t}_{i+1})}^{(\,j)}| {\boldsymbol{Z}}{({t}_{i})}^{(\,j)}\right)\\ &=&{{{\mathcal{N}}}}\left({\boldsymbol{Z}}{({t}_{i+1})}^{(\,j)}| {\boldsymbol{Z}}{({t}_{i})}^{(\,j)}+{f}_{\theta }({\boldsymbol{Z}}{({t}_{i})}^{(\,j)}){{\Delta }}t,{\sigma }_{\theta }({\boldsymbol{Z}}{({t}_{i})}^{(\,j)}){\sigma }_{\theta }^{T}({\boldsymbol{Z}}{({t}_{i})}^{(\,j)}){{\Delta }}t\right)\\ &=&\frac{1}{{(2\pi {{\Delta }}t)}^{d/2}\sqrt{\det ({\sigma }_{\theta }{\sigma }_{\theta }^{T})}}\exp \left\{-\frac{1}{2{{\Delta }}t}{[{\boldsymbol{Z}}{({t}_{i+1})}^{(\,j)}-{\boldsymbol{Z}}{({t}_{i})}^{(\,j)}-{f}_{\theta }({\boldsymbol{Z}}{({t}_{i})}^{(\,j)}){{\Delta }}t]}^{T}\right.\\ &&\left.{({\sigma }_{\theta }{\sigma }_{\theta }^{T})}^{-1}[{\boldsymbol{Z}}{({t}_{i+1})}^{(\,j)}-{\boldsymbol{Z}}{({t}_{i})}^{(\,j)}-{f}_{\theta }({\boldsymbol{Z}}{({t}_{i})}^{(\,j)}){{\Delta }}t]\right\},\end{array}$$

where we use the short form ${\sigma }_{\theta }={\sigma }_{\theta }({\boldsymbol{Z}}{({t}_{i})}^{(\,j)})$ and det denotes the determinant of a matrix.

Taking the logarithm of the above equation, we obtain

$$\begin{array}{rcl}&&\log p\left({\boldsymbol{Z}}{({t}_{i+1})}^{(\,j)}| {\boldsymbol{Z}}{({t}_{i})}^{(j)}\right)\\ &=&-\frac{1}{2}\log \det ({\sigma }_{\theta }{\sigma }_{\theta }^{T})-\frac{{{\Delta }}t}{2}{\left(\frac{{\boldsymbol{Z}}{({t}_{i+1})}^{(j)}-{\boldsymbol{Z}}{({t}_{i})}^{(j)}}{{{\Delta }}t}-{f}_{\theta }({\boldsymbol{Z}}{({t}_{i})}^{(j)})\right)}^{T}\\ &&{({\sigma }_{\theta }{\sigma }_{\theta }^{T})}^{-1}\left(\frac{{\boldsymbol{Z}}{({t}_{i+1})}^{(j)}-{\boldsymbol{Z}}{({t}_{i})}^{(j)}}{{{\Delta }}t}-{f}_{\theta }({\boldsymbol{Z}}{({t}_{i})}^{(j)})\right)+\,{{\mbox{constant}}}\,.\end{array}$$

As a result, we may obtain the loss function by maximizing the log-likelihood of the training data

$$\begin{array}{rcl}&&{{{{\rm{loss}}}}}_{{{{\rm{MLE}}}}}\\ &=&\frac{1}{{N}_{t}M}\mathop{\sum }\limits_{i=1}^{{N}_{t}}\mathop{\sum }\limits_{j=1}^{M}\left(\frac{1}{2}\log \det ({\sigma }_{\theta }{\sigma }_{\theta }^{T})+\frac{{{\Delta }}t}{2}{\left(\frac{{\boldsymbol{Z}}{({t}_{i+1})}^{(\,j)}-{\boldsymbol{Z}}{({t}_{i})}^{(\,j)}}{{{\Delta }}t}-{f}_{\theta }({\boldsymbol{Z}}{({t}_{i})}^{(\,j)})\right)}^{T}\right.\\ &&\left.{({\sigma }_{\theta }{\sigma }_{\theta }^{T})}^{-1}\left(\frac{{\boldsymbol{Z}}{({t}_{i+1})}^{(\,j)}-{\boldsymbol{Z}}{({t}_{i})}^{(\,j)}}{{{\Delta }}t}-{f}_{\theta }({\boldsymbol{Z}}{({t}_{i})}^{(\,j)})\right)\right).\end{array}$$

The total loss is then

19 $$\begin{array}{r}{{{\rm{loss}}}}={{{{\rm{loss}}}}}_{{{{\rm{MLE}}}}}+\rho \,{{{{\rm{loss}}}}}_{{{{\rm{Rec}}}}},\end{array}$$

where *ρ* is a parameter to balance the accuracy of the learned dynamics and the error from reconstruction. In our computation, we first train the loss function (19) with *ρ* = 0.01. After some training steps, we fix the encoder part (17) and the decoder part (18) of the neural network, and train loss_MLE_ (*ρ* = 0) to fine-tune accuracy of the stochastic dynamics. We use the Adam optimizer for training. The overall implementation, including the network architectures and loss computation, is shown in Supplementary Fig. 2.

### Data preparation

#### Simulation data

We used a Brownian dynamics approach to simulate linear, touching-bead chains as polymer chains in a planar elongational flow (Fig. 2, left). Each polymer chain consisted of *N* = 300 (*D* = 3*N*) beads with diameter *r* at positions of bead *i* **r**_*i*_, connected by *N* − 1 rigid rods of length *b* = *r* = 10 nm. The governing stochastic differential equation was obtained by considering the following forces acting on the system: excluded volume, constraint, Brownian and hydrodynamic.

The excluded volume potential characterizes the short-range repulsions between beads and can be described by

$${E}^{{\mathrm{ev}}}=-\mathop{\sum }\limits_{i,\,j}^{N}\mu {r}_{ij}\quad \,{{\mathrm{if}}}\,\,{r}_{ij} < r\,\,{{\mathrm{nm}}}\,$$

where *μ* = 35 pN has been demonstrated to result in a low frequency of chain crossings^47^. The constraint force is given by

$${{{{\bf{F}}}}}_{i}^{{\mathrm{c}}}={T}_{i}{{{{\bf{b}}}}}_{i}-{T}_{i-1}{{{{\bf{b}}}}}_{i-1}$$

where **b**_*i*_ is the unit vector of bond *i* and *T*_*i*_ is the tension in rod *i* that imposes constant bond length. The Brownian forces are random forces that satisfy the fluctuation-dissipation theorem, represented as

$$\left\langle {{{{\bf{F}}}}}_{i}^{{\mathrm{br}}}(t)\right\rangle =0\quad \,{{\mathrm{and}}}\,\quad \left\langle {{{{\bf{F}}}}}_{i}^{{\mathrm{br}}}(t){{{{\bf{F}}}}}_{j}^{{\mathrm{br}}}(t)\right\rangle =\frac{2{k}_{{\mathrm{B}}}T\zeta {{{\bf{I}}}}{\delta }_{ij}}{{{\Delta }}t}$$

where *δ*_*ij*_ is the Kronecker delta, **I** is the identity matrix and Δ*t* is the simulation time step. By neglecting hydrodynamic interactions between the beads, the drag force on the *i*th bead is

$${{{{\bf{F}}}}}_{i}^{{\mathrm{d}}}=-\zeta \left({{\bf{u}}}({{\boldsymbol{r}}}_{{{{\bf{i}}}}})-\frac{\,{{\mathrm{d}}}{{{{\bf{r}}}}}_{{{{\bf{i}}}}}}{\,{{\mathrm{d}}}t}\right)$$

where *ζ* ≈ 3π*ηr* is the drag coefficient of a single bead, *η* is the solvent viscosity and **u**(**r**_**i**_) is the unperturbed solvent velocity.

Due to the small mass of the beads, it is common to neglect inertial effects and set the sum of forces on the beads to be zero. Hence, the Langevin equation that describes the motion of each bead along the chain is

$$\frac{\,{{\mathrm{d}}}{{{{\bf{r}}}}}_{{{{\bf{i}}}}}}{\,{{\mathrm{d}}}t}={\bf{u}}({{\boldsymbol{r}}}_{{{{\bf{i}}}}})+\frac{1}{\zeta }\left({{{{\bf{F}}}}}^{{\mathrm{ev}}}+{{{{\bf{F}}}}}^{{\mathrm{c}}}+{{{{\bf{F}}}}}^{{\mathrm{br}}}\right)$$

We employed a predictor-corrector scheme^48^ to determine the position of each bead at every time step. The enforcement of rigid rod constraints leads to a system of nonlinear equations for the rod tensions *T*_*i*_, which we solved for using Newton’s method.

For each simulation run, the polymer chain was allowed to equilibrate for 10^4^ *τ*_d_, with *τ*_d_ = *b*^2^*ζ*/*k*_B_*T* being the characteristic rod diffusion time. During equilibration, the chain would adopt random configurations as governed by thermal fluctuations. At *t* = 0, the chain was subjected to a planar elongational flow of the form ${{{\bf{u}}}}({{{{\bf{r}}}}}_{{{{\bf{i}}}}})=\dot{\epsilon }(\hat{{{{\bf{x}}}}}-\hat{{{{\bf{y}}}}})\cdot {{{{\bf{r}}}}}_{{{{\bf{i}}}}}$, where $\dot{\epsilon }$ is the strain rate and $\hat{{{{\bf{x}}}}}$ and $\hat{{{{\bf{y}}}}}$ are unit vectors parallel to the *x* and *y* axes, respectively. The simulations were run until *t* = 10^4^ *τ*_d_, using a time step of Δ*t* = 5 × 10^−4^ *τ*_d_. To generate training data, we simulated 610 stretching trajectories. To test the predictions, we simulated 500 stretching trajectories each for 3 different initial chain configurations, which were deliberately selected from 3 vastly different trajectories. For each trajectory, we obtain the (*x*, *y*, *z*) positional coordinates **r**_**i**_ of *N* beads every 10 *τ*_d_. Given the observation data, we can get the chain extension (Fig. 2). We note that time reported hereon is in units of *τ*_d_. Although the dataset in this work is generated based on known equations, it should be highlighted that our machine learning approach for constructing the reduced dynamical model and all resulting consequences are independent of the microscopic model used in the simulations, with only the positional coordinates as inputs into the S-OnsagerNet. In other words, the approach used is purely data driven and can therefore be generally applied to other non-equilibrium problems.

#### Experimental data

We provide the details of the experimental validation of the S-OnsagerNet results.

##### Experiments on electrokinetic stretching of DNA

To collect the experimental data leading to Fig. 6, we needed to create an automated single-molecule stretching trap. We describe the essential features of the experiments, while the reader can find additional details about the trap and the material preparation methodology in ref. ^49^. The polymer samples used for this study were T4 phage double-stranded DNA (165.6 kbp, Nippon Gene), chosen for high monodispersity and ready availability. The DNA was diluted in buffer solution and fluorescently labeled (YOYO-1) to aid visualization.

The trap was based on a microfluidic cross-slot channel device with a wide central chamber, splitting into 40-μm-wide channels in each of the four cardinal directions (Supplementary Fig. 3). Each of the four channels was terminated by a macroscopic reservoir, in which we pipetted the DNA sample and inserted platinum wire electrodes. These electrodes were connected to a computer-controlled analog voltage source, such that the north and south electrodes were grounded, but east and west were positively biased *V*_0_ = +30 V. This electric quadrupole arrangement set up a potential well in the north–south direction and a potential hill in the east–west direction. The saddle point was located in the central chamber.

In aqueous solution, DNA is naturally negatively charged, and in the microfluidic device, molecules drifted electrokinetically from the north and south reservoirs towards east and west. At the saddle point, the east–west potential hill could be exploited to stretch a DNA molecule, but being an unstable equilibrium point, molecules would approach its location and slow down, but could not remain there for long observation times without external intervention.

To actively trap and observe single DNA molecules at the saddle point, the microfluidic electrokinetic device was placed on an inverted fluorescence microscope (Nikon Eclipse Ti2U) with a ×60 oil-immersion objective (1.4 numerical aperture). A live fluorescence image was captured by a scientific complementary metal–oxide–semiconductor camera (Teledyne Photometrics Prime 95b) at 50 ms intervals and sent to a desktop computer that analyzed the incoming images in real time. The image analysis included a fast clean-up step (detailed in the next section) that removed background noise and stray ‘passer-by’ molecules/fragments, followed by a step to calculate the intensity centroid of the target molecule and its projected length. The displacement *x* of the molecule’s centroid from the saddle point was input to a proportional feedback loop that output a voltage tilt Δ*V* ∝ *x*, which was then superimposed on the east–west electrode biases, such that Δ*V* = *V*_0_ ± *Gx*. Setting *G* = 2.2 V μm^−1^ confined the DNA centroid to within 1 μm of the saddle point even while a molecule stretched. This feedback process for tracking and trapping DNA molecules was automated through a custom LabVIEW program.

In a single stretch experiment, the platform was programmed to actively search for a molecule to trap as they flowed through the central chamber. Once a molecule was trapped, *V*_0_ was set to zero temporarily to allow the molecule to relax into an unstretched, equilibrium state over 10 s (chosen based on experience from preliminary experiments). After this relaxation period, recording began with images being streamed to the computer’s solid-state drive. With every image, the associated centroid coordinates, projected length, voltages and other parameters were logged in real time. *V*_0_ was then reset to +30 V, which re-established the east–west potential hill and stretched the DNA molecule. By monitoring the projected length history, the platform could recognize when the molecule was fully stretched, and in response stop the recording and release the molecule to escape naturally towards east or west. Using this protocol, we were able to capture a diverse set of stretching trajectories, including the dumbbell and folded conformations that were selected for analysis in Fig. 6.

##### Real-time image processing

A DNA molecule deforms over time, and the purpose of this section is to elucidate our method used to extract sequential image snaps that can be used to capture the DNA unfolding. To capture the exact location of the DNA molecule, we devised an algorithm to classify pixels according to the density of a pixel’s immediate surrounding. The algorithm exploited the fact that the targeted molecule would very likely be centered in the tracking region of interest (ROI), and this was used to discriminate against stray particles during tracking. To make this algorithm fast enough for real time, the algorithm operated only on the binarized version of the raw image, after application of a threshold. The result was a binary image containing the targeted DNA pixels at the exclusion of pixels belonging to noise speckles and stray particles. See Supplementary Fig. 4 as an illustrated example of the real-time image processing pipeline.

One would imagine that calculating the intensity centroid is a natural method for defining the molecule location and tracking it across image frames in time. However, doing so with the raw DNA images is problematic for the two following reasons.

First, background noise from the camera biases the centroid towards the center of the frame. This occurred even though we subtracted a pre-recorded background frame. The problematic noise in this case arises from pixel shot noise (electronic, photon) and pixel readout, and manifests as low-intensity sparkles in the image. One possible solution is to increase the excitation intensity, but this caused the DNA to photocleave much more readily, too often breaking into fragments before stretching out fully.

Second, while a trapped DNA molecule was being stretched, stray molecules continued to drift into the camera’s field of view, which biased the centroid towards these stray molecules. This problem was partially solved by calculating the centroid from a smaller ROI that was just large enough to cover the fluctuating motion of a single molecule. This ROI was software based and centered on the molecule centroid for tracking, that is implemented as a subset of the image array. The reduced ROI size avoided most of the stray particles, but it was still common for some to enter the tracking ROI during a trap–stretch cycle.

##### Data selection

Among the stretching trajectories collected, a few were selected for further analysis. Specifically, examples of molecules adopting the ‘dumbbell’ and ‘folded’ conformations during the stretching process with similar initial extension lengths were chosen to benchmark to human labeling. The trajectories of the conformations as the molecules were stretched were plotted in the reduced coordinates space (Fig. 6). When in the fully stretched stable state, the DNA molecules still undergo conformational fluctuations due to Brownian motion, which result in fluctuations in the reduced coordinate space. Images of molecules in this stable state were analyzed to obtain the fluctuations in *Z*_1_, *Z*_2_ and *Z*_3_, as plotted in Fig. 6. The method to extract the learned thermodynamic coordinates *Z*_2_, *Z*_3_ from experimental images are described next.

##### Extracting reduced coordinates from filtered images

Different from the simulated data described earlier, given a filtered image, it is more challenging to obtain in a robust way a sequence of ordered coordinates representing the location of each part of the DNA molecule. However, identifying the two end points is easier. Thus, we first find the coordinates of the two end points and also the center of mass (weighted by intensity) of all illuminated pixels (Supplementary Fig. 5). Then, we estimate *Z*_2_, *Z*_3_ by computing the end-to-end distance and foldedness as defined in Fig. 3, which are shown to strongly correlate with *Z*_2_ and *Z*_3_, respectively. These computations require only the relative position vectors of the two end points with respect to the center of mass (***r***_1_ = (*r*_1,*x*_, *r*_1,*y*_, 0) and ***r***_*N*_ = (*r*_*N*,*x*_, *r*_*N*,*y*_, 0)). The third coordinate is set to zero because the DNA molecule is confined to have limited motion in this direction. The center of mass ***r***_cm_ is computed by

$$\begin{array}{r}{{\boldsymbol{r}}}_{{{{\rm{cm}}}}}=\frac{1}{\bar{I}}\mathop{\sum}\limits_{m,n}{u}_{m,n}{I}_{m,n},\end{array}$$

where $\bar{I}={\sum }_{m,n}{I}_{m,n},{u}_{m,n}$ is the spatial position of the (*m*, *n*)-pixel and *I*_*n*,*m*_ is the intensity of the image at pixel (*m*, *n*). This allows for the computation of ***r***_1_ and ***r***_*N*_ (Supplementary Fig. 5). According to the definition of end-to-end distance and foldedness and their linear correlation with *Z*_2_, *Z*_3_ (Fig. 3), we may compute the reduced coordinates as

$$\begin{array}{rcl}{Z}_{1}&=&{C}_{1}L,\\ {Z}_{2}&=&{C}_{1}{C}_{2}({r}_{1,x}-{r}_{N,x}),\\ {Z}_{3}&=&{C}_{1}[{C}_{3}({r}_{1,x}+{r}_{N,x})+{C}_{4}],\end{array}$$

where *L* is the extension length scale of the experimental data, and the parameters *C*_1_, *C*_2_, *C*_3_ and *C*_4_ are scaling parameters to account for the change in molecule configuration properties (for example, length scales) from simulation to experimental data. In particular

- *C*_1_ is obtained by dividing the average extension of unfolding simulated data by that of experimental data.
- *C*_2_ is the relationship between *Z*_2_ and end-to-end distance (Fig. 3b) and is obtained by dividing the average value of *Z*_2_ in simulated data by that of the end-to-end distance.
- *C*_3_ and *C*_4_ is obtained by the relationship between the *Z*_3_ and foldedness. We use least squares method to get *C*_3_ and *C*_4_ (Fig. 3e).

The method of extraction differs in the plot of Fig. 6j. Here we consider only the stretched state, for which we can extract 300 coordinates (assuming the third coordinate *z* = 0) equally spaced along the stretched polymer. A scaling along the *x* axis is performed so that the average full extension of the experimental polymer data matches that of the simulation data. These coordinates are then fed into the trained PCA-ResNet to extract the *Z*_2_ and *Z*_3_ values.

### Polymer dynamics analysis

In this section, we detail the modeling of the polymer unfolding problem using the S-OnsagerNet.

#### Accurate prediction of the statistics of unfolding

In our training process, we have 610 training trajectories and 110 testing trajectories. Note that although the dataset is generated by known yet complex microscopic equations, our approach does not require, nor rely on, the knowledge of these equations. The chain extension evolution of the training data with different initial chain are shown in Supplementary Fig. 6a (black) and the test data are shown in Supplementary Fig. 6e (black). After training the model, we predict the extension of the polymer (red). We can see the extension of the polymer can be predicted well. We also compute the mean (Supplementary Fig. 6b,f), standard derivation (Supplementary Fig. 6c,g) and the distribution of unfolding time (Supplementary Fig. 6d,h) of the training and test results. We also compute the error of the mean (relative *L*^2^ error), standard derivation and the probability distribution of unfolding time of the training and test results in Supplementary Table 1. We observe that our model successfully captures the statistical behavior of a polymer stretching with only a three-variable dynamical system.

#### Interpreting the learned closure coordinates

Supplementary Fig. 7 shows the evolution of chain extension and the two learned closure coordinates with time for the training data. The trajectories are colored by unfolding time. Based on the unfolding times, we observe that chains with similar initial extensions (determined by the *y* intercept, that is, *Z*_1_ at *t* = 0) can take vastly different times to stretch; hence, it is not sufficient to consider only chain extension for the purposes of predicting dynamics. However, the successful prediction of the statistics of unfolding dynamics implies that *Z*_2_ and *Z*_3_ capture crucial information of the system. Thus, we seek to gain some physical understanding of the learned closure coordinates *Z*_2_ and *Z*_3_.

Here we provide details on the analysis of the closure coordinates (*Z*_2_, *Z*_3_) that characterize the stochastic evolution of the extension length (*Z*_1_), Recall that *Z*_2_, *Z*_3_ are deterministic functions of the microscopic configuration ***X***, and the functions are approximated by a trained PCA-ResNet. A useful property we can exploit is differentiability of the neural network. We can ask: given a certain configuration ***X***, what kind of small perturbations to *X* will most drastically increase or decrease the value of *Z*_2_ and *Z*_3_? In other words, we can consider perturbations in the directions of ±∂*Z*_2_/∂***X*** and ±∂*Z*_3_/∂***X***, respectively. We first analyze the closure coordinate *Z*_2_. From Fig. 3a, we observe that perturbations in the direction of *Z*_2_ tend to change the end-to-end distance in the elongational axis (distance between the first and the last bead in the polymer chain along the elongational direction, that is |*r*_*N*,*x*_ − *r*_1,*x*_|), where *r*_*j*,*i*_ is the *i*th coordinate of the *j*th bead in the chain (cyan, *Z*_2_ = 0.453; blue, *Z*_2_ = 0.194; black, *Z*_2_ = 0.323). We confirm this hypothesis by visualizing the correlation of the end-to-end distance and the magnitude of *Z*_2_ in Fig. 3b,c. In general, we observe that as |*Z*_2_| decreases, the distance between the chain ends (marked by red points in the figure) decreases. Thus, we can interpret the first learned closure coordinate as an indicator of end-to-end distance.

We proceed with a similar analysis for the other closure coordinate *Z*_3_. Figure 3d shows a given chain configuration and perturbations in the positive and negative directions of $\frac{\partial {Z}_{3}}{\partial {\boldsymbol{X}}}$ (cyan, *Z*_3_ = 7.903; blue, *Z*_3_ = 1.595; black, *Z*_3_ = 4.749). Here we observe that the end-to-end distance is largely unchanged, but the degree of foldedness of the chain in the elongational axis of the flow (*x* direction) appears to change. This leads us to hypothesize that the second learned coordinate represents a degree of foldedness with respect to the elongational flow. As a measure of the degree of foldedness of a chain, we compute |*r*_1,*x*_ + *r*_*N*,*x*_|. During data pre-processing, the chain is centered such that its center of mass is 0. Hence, if |*r*_1,*x*_ + *r*_*N*,*x*_| is small, the polymer is symmetric around zero in the elongational *x* direction and tends to be in the elongated state. If |*r*_1,*x*_ + *r*_*N*,*x*_| is large, the polymer is likely to be in the folded state. We plot |*r*_1,*x*_ + *r*_*N*,*x*_| as a function of |*Z*_3_| for all configurations in the training dataset in Fig. 3e. The strong correlation between |*r*_1,*x*_ + *r*_*N*,*x*_| and |*Z*_3_| supports our interpretation that the second learned closure coordinate is an indicator of the degree of foldedness in the elongational direction. To demonstrate this, we plot in Fig. 3f visualizations of different chains with a range of |*Z*_3_| values, with the chain ends marked by red points. As |*Z*_3_| decreases, we observe tat the chains generally shift from the folded to the elongated state. We note that the degree of foldedness is sufficiently described solely by considering the projection of chain coordinates onto the elongational axis of the flow, as the flow is stable in the compressional axis and thus the degree of foldedness is primarily relevant to the unstable elongational axis that drives the unfolding process.

#### Advancing classification methods for polymer stretching

With the new understanding of the closure variables, we now consider how our results improve the current understanding of polymer chain dynamics. In the landmark experimental study of dilute polymer chains stretching under elongational flow, the molecules were categorized into seven different conformations and the dynamics of dominant conformations were analyzed^25^. Specifically, it was found that chains in the ‘folded’ conformation (which is one of the seven categories) took the longest time to stretch, while chains in the dumbbell conformation stretched relatively quickly (Supplementary Fig. 8).

Our analysis shows that instead of a categorical labeling, it is perhaps more useful to characterize the stretching dynamics of a polymer by three numbers representing the generalized coordinates: *Z*_1_ (extension length), *Z*_2_ (related to end-to-end distance) and *Z*_3_ (related to foldedness in the flow direction). Our results show that these are sufficient to predict the dynamics of the chain extension. We show that this characterization is largely consistent with previous categorical ones, but improves on them in some intermediate cases. In Supplementary Fig. 9, we plot the values of the low-dimensional coordinates |*Z*_2_| and |*Z*_3_| of different chains with initial dumbbell and folded configurations at selected chain extension *Z*_1_ values, colored by the predicted unfolding times. We observe segregation between the folded and dumbbell configurations in the *Z*_2_–*Z*_3_ space, indicating that the qualitative differences between different conformations can be captured by our characterization. In general, the folded chains take a longer time to stretch compared with the dumbbell chains. This is consistent with experimental and computational observations reported in the literature^25–27^. However, we highlight that the region with high |*Z*_2_| and low |*Z*_3_| values encompasses a mix of folded and dumbbell chains with similar unfolding times. Therefore, while the broad categorization scheme allows for coarse discrimination of the stretching dynamics, the qualitative classification does not allow for finer predictions. Instead of classifying the stretching trajectories based on qualitative, human judgement of chain conformation during the process, we present a robust, quantitative approach to interpreting the stretching dynamics that involves consideration of the initial chain configuration in reduced dimensions.

#### Free-energy landscape analysis

We now provide details on the analysis of the free-energy landscape. We begin with an important technical note. Our learned GSOP following equation (5) is in general not guaranteed to be a gradient system, unless *W*(***Z***) = 0 and *M*(***Z***) = *I*. However, as the drift term $f({\boldsymbol{Z}}\,)={({f}_{1}({\boldsymbol{Z}}\,),{f}_{2}({\boldsymbol{Z}}\,),{f}_{3}({\boldsymbol{Z}}\,))}^{T}=-(M({\boldsymbol{Z}}\,)+W({\boldsymbol{Z}}\,))\nabla V({\boldsymbol{Z}}\,)$, the stationary points of *V* are also critical points of the dynamics (*f*(*Z*) = 0). The saddle points of *V* are the saddle foci of the non-gradient dynamics $\dot{Z}=f({\boldsymbol{Z}}\,)$. For simplicity, we refer to a saddle point of *V* and saddle focus of *f* interchangeably.

We compute the critical points by numerically solving ∇*V*(*Z*) = 0 with the BFGS method from different initial conditions. We obtain four critical points: (247.15, 1.701, 0.193) (yellow star), (247.29, −1.700, −0.166) (blue square), (87.858, −0.041, −4.269) (cyan triangle) and (85.887, −0.050, 4.126) (purple diamond), which are shown in Fig. 4. The Jacobian matrix of the drift term is then computed

$$J(\,f\,)=\left(\begin{array}{ccc}{\partial }_{{Z}_{1}}{f}_{1}&{\partial }_{{Z}_{2}}\,{f}_{1}&{\partial }_{{Z}_{3}}\,{f}_{1}\\ {\partial }_{{Z}_{1}}{f}_{2}&{\partial }_{{Z}_{2}}\,{f}_{2}&{\partial }_{{Z}_{3}}\,{f}_{2}\\ {\partial }_{{Z}_{1}}{f}_{3}&{\partial }_{{Z}_{2}}\,{f}_{3}&{\partial }_{{Z}_{3}}\,{f}_{3}\\ \end{array}\right).$$

We calculate the eigenvalues and eigenvectors of *J* at the four critical points. It has three eigenvalues with negative real parts at the yellow and blue points, so these are the stable points (stable nodes). We name them ***Z***_stable,1_ and ***Z***_stable,2_. For the cyan triangle and magenta diamond points, *J* has one eigenvalue with positive real part, and two with negative real parts. These points are saddle points (saddle focus) of index 1. We call these two points ***Z***_saddle,1_ and ***Z***_saddle,2_. The direction of the eigenvector corresponding to the eigenvalue with positive real part is the unstable manifold, along which the trajectories escape from the saddle. On the other hand, the span of the real and imaginary parts of the other eigenvector (one must be a complex conjugate of the other) constructs the stable manifold, along which trajectories are attracted to the saddle.

The local behavior of the learned potential ***V***(***Z***) around its critical points characterize the typical fluctuations around them. There lies important physical meaning that we can probe. For example, let us exploit automatic differentiation of neural networks and expand *V*(*Z*) in a Taylor series around *Z*_stable,1_ (fully stretched state), corresponding to (247.15, 1.701, 0.193). Neglecting small terms, we obtain the approximate formula

20 $$\delta V\approx 153.1{(\delta {Z}_{1}-1.54\delta {Z}_{2})}^{2}+205.5\delta {Z}_{2}^{2}+36.96\delta {Z}_{3}^{2},$$

where $\delta {Z}_{i}={Z}_{i}-{[{Z}_{{{{\rm{stable}}}},1}]}_{i}$ is the fluctuations in the thermodynamic variables. Now, assuming that the distribution of states around ***Z***_stable_ follows a Boltzmann distribution ${\boldsymbol{Z}} \sim \exp [-V({\boldsymbol{Z}}\,)/{k}_{{\mathrm{B}}}T]$ (here we assume that *M* ≈ *I*) where |*σ*|^2^ ∝ *k*_B_*T*, the typical small fluctuations δ*V* is proportional to *k*_B_*T*. In other words, equation (20) is approximate ‘isotherms’ that captures the form of typical fluctuations. For example, we can infer from the formula that typical fluctuations of the extension length (*Z*_1_) and the end-to-end distance (*Z*_2_) are highly correlated. This is sensible, as in the fully stretched state, these two quantities are expected to change simultaneously. In Fig. 4g–i, we confirm these correlations.

Similarly, one can also expand *V*(***Z***) around a saddle point (fully folded state) ***Z***_saddle,2_. We obtain the formula

$$\delta V\approx 102.96\delta {Z}_{1}^{2}-31.13{(\delta {Z}_{2}-0.255\delta {Z}_{3})}^{2}+24.16\delta {Z}_{3}^{2},$$

Here we immediately observe that to escape the saddle point (lowering the energy), one should increase end-to-end distance (*Z*_2_) and decrease foldedness (*Z*_3_). This approximately aligns with the unstable manifold described above, and forms the basis of our control protocols described in the main text.

#### Selection of the dimension of the reduced coordinates

In this part, we describe how we arrived at the selection of a three-variable reduced coordinate space. We tested various different numbers of reduction dimensions (*d* = 2, 3, 4) and the relative errors of predictions are summarized in Supplementary Table 1. We observed that going to a higher dimension (*d* = 4) did not result in noticeable gains. In fact, increasing dimension may cause increasing optimization error and hence worse results in standard deviation. Going to a lower dimension (*d* = 2) resulted in increased prediction error in general.

Interestingly, we can formulate a physical argument that suggests that a two-dimensional system (with only one additional closure coordinate) is not a suitable reduced model for the polymer dynamics we study. The argument is based on index theory for two-dimensional dynamical systems^50^.

Let us assume for the sake of contradiction that the projected free-energy landscape of our learned three-dimensional system into *Z*_1_–*Z*_2_ plane is the free energy of a system in two-dimensional (Supplementary Fig. 10). As *Z*_1_ is the polymer extension, we expect that

1. There exists two stable states at large *Z*_1_ corresponding to the fully extended state. There are two of them due to reflection symmetry in the flow direction. Around this *Z*_1_ value, all trajectories in the reduced space should converge to one of the stable steady states.
2. There cannot be saddle points with the same *Z*_1_ value, as it is close to the maximal extended chain length.

These conditions are enough to imply a contradiction in the following way. The simple concept we use from index theory is the definition of the index of a closed curve in the phase space of a two-dimensional dynamical system (see Ch. 6.8 in ref. ^50^ for details). Let *Γ* be a closed curve in $${{\mathbb{R}}}^{2}$$, and consider a dynamical system $$\dot{{\boldsymbol{z}}}=f({\boldsymbol{z}})$$$$={(\;{f}_{1}({\boldsymbol{z}}),{f}_{2}({\boldsymbol{z}}))}^{T},{\boldsymbol{z}}={({{\boldsymbol{z}}}_{1},{{\boldsymbol{z}}}_{2})}^{T}\in {{\mathbb{R}}}^{2}$$. The index of *Γ* with respect to the dynamics *f* is defined as

21 $$\begin{array}{r}{I}_{{{\varGamma }}}(\,f\,):= \frac{1}{2\uppi }\displaystyle{\oint }_{{{\varGamma }}}\frac{{f}_{1}d{f}_{2}-{f}_{2}d{f}_{1}}{{f}_{1}^{\,2}+{f}_{2}^{\,2}}.\end{array}$$

Intuitively, this is the sum of the angles of the force vectors across the curve *Γ*.

From index theory, we know that the index of a closed curve must equal to the sum of indices of critical points it encloses. Moreover, the index of a stable critical point is +1, and the index of a saddle is −1. Now, we draw a curve enclosing the two stable points as shown in Supplementary Fig. 10. By condition (1) above, the vector fields should point inwards towards the interior of the curve, thus we can show (from equation (21)) that the index of this curve is +1. However, it can only enclose stable critical points due to condition (2), and consequently its index is at least +2. Thus we arrive at a contradiction, showing that the dynamical landscape in Supplementary Fig. 10 is not possible in two dimensions.

The only remaining possibility is that *Z*_2_ does not distinguish the two symmetric stable extended states. However, in this case to allow a saddle point (which is the key to inducing heterogeneity in unfolding), we must have another stable steady state at a different *Z*_1_ value. This is unlikely from a physical viewpoint for non-self-interacting polymers, as we expect the only asymptotically stable state to be the stretched, fully extended state.

### The impact of dataset size on predictive accuracy of S-OnsagerNet

To study the impact of the number of training data on the computational accuracy of the model, the original training dataset containing 610 trajectories was split into two subsets containing 25% and 75% of the data. A third subset was produced by selecting at random 50% of the data from the original dataset. The three datasets and the learned potential landscapes are presented in Supplementary Fig. 11. The 25% and 75% datasets have no common trajectories, while the 50% dataset contains some trajectories from each of the other two datasets. The quantitative results reported in Supplementary Table 2, where the trained models are used to predict 500 unseen trajectories with fast, medium and slow unfolding times, suggest that with a smaller amount of training data, the S-OnsagerNet model loses some predictive accuracy. However, the more important factor is the diversity of the data contained in the training datasets. The full dataset has a high proportion of trajectories with fast, followed by middle (slower) and then slow unfolding times; however, it is the most balanced of all the training datasets (25%, 50%, 75%, 100%), in addition to containing the most data. Training with the 25% dataset, which contains the largest proportion of trajectories with fast unfolding times results in the lowest *L*^2^ error, but the model overfits to that type of trajectory, and has the largest *L*^2^ error for the medium and slow unfolding times. However, the 50% dataset is the most balanced of the reduced datasets, which is reflected in the relative *L*^2^ errors. It can also be observed in Supplementary Fig. 11 (bottom) that the potential landscapes resulting from training with smaller datasets do not contain a clear stagnation (saddle) point. Overall, for training datasets with different numbers of trajectories and their respective proportions of fast, medium and slow trajectories, the results, both in terms of prediction error and potential landscape characteristics, suggest that the model is relatively robust, and the amount of training data cannot be reduced much without affecting predictive performance.

### Spatial epidemics analysis

In this section, we provide details of our analysis method for an alternative application of S-OnsagerNet—modeling the macroscopic dynamics of the spread of epidemics. This highlights the general applicability of our method.

We focus on the most well-known model for disease spread in a spatial domain—the spatial SIR model^32^. Let us consider a two-dimensional square domain (representing a city, say) discretized into *n* × *n* sectors. We use *I*_*i*,*j*_ and *S*_*i*,*j*_ to represent the number (density) of infective and susceptible individuals at spatial location (*i*, *j*). The basic mechanism of the model is as follows: each infective individual may infect a susceptible individual in the same spatial location. At the same time, each infective individual recovers (or is removed) at a rate, after which they are no longer infective. Finally, both infective and susceptible individuals move on the spatial domain randomly. Mathematically, the temporal evolution of *I*, *S* (understood as *n* × *n* matrices, or length *n*^2^ vectors) are governed by the following dynamics

22 $$\begin{array}{rcl}{\dot{S}}_{i,\,j}&=&-\beta {I}_{i,\,j}{S}_{i,\,j}+\frac{\delta }{{\delta }_{x}^{2}}\left({S}_{i-1,\,j}-2{S}_{i,\,j}+{S}_{i+1,\,j}\right)\\ &&+\frac{\delta }{{\delta }_{y}^{2}}\left({S}_{i,\,j-1}-2{S}_{i,\,j}+{S}_{i,\,j+1}\right)+\sigma {\dot{B}}_{1}(t),\\ {\dot{I}}_{i,\,j}&=&\beta {I}_{i,\,j}{S}_{i,\,j}-\gamma {I}_{i,\,j}+\frac{\delta }{{\delta }_{x}^{2}}\left({I}_{i-1,\,j}-2{I}_{i,\,j}+{I}_{i+1,\,j}\right)\\ &&+\frac{\delta }{{\delta }_{y}^{2}}({I}_{i,\,j-1}-2{I}_{i,\,j}+{I}_{i,\,j+1})+\sigma {\dot{B}}_{2}(t).\end{array}$$

As usual, the dot denotes time derivative. The parameter *β* is a measure of the transmission efficiency of the disease from infectives to susceptibles, and 1/*γ* is the life expectancy (or expected recovery time) of an infective. The constant *δ* is the diffusion coefficient, and this term in the equation models the spatial movement of individuals as a diffusion process over the domain. The last terms of the equation models the stochastic fluctuations of the number densities, with *σ* as the noise intensity. The parameters *δ*_*x*_ and *δ*_*y*_ are the spatial discretization sizes in the two spatial directions. In our simulations, we take *n* = 16, *β* = 0.3, *γ* = 0.13, *δ* = 0.5, *σ* = 0.03 and ${\delta }_{x}={\delta }_{y}=\frac{2}{3}$. Equation (22) governs the microscopic dynamics of disease spread, and non-trivial outcomes can result from different initial spatial configurations and parameters (for example, infection rate, recovery rate). See Extended Data Fig. 1.

While this microscopic model and its variants has been subject to intense study (see ref. ^32^ and references therein), a macroscopic understanding of the dynamics of disease spread is challenging due to the complex spatial interactions. For example, one may be interested to model the dynamics of average (or total) number of infective and susceptible individuals over the spatial domain. Observe in Extended Data Fig. 1 that configurations with identical initial spatial averages of infective and susceptible individuals can have drastically different subsequent evolution. More precisely, one spatial configuration Extended Data Fig. 1a can lead to initial disease spread (epidemic), where the mean number of infected individuals initially increases sharply, whereas another spatial configuration in Extended Data Fig. 1b leads to the disease dying out monotonically. Thus, it is of interest to develop a thermodynamic description to capture and elucidate the driving factors of such variations.

#### Modeling the thermodynamics of the spatial SIR model using S-OnsagerNet

Following our framework, we now set the macroscopic variables of interest as the respective spatial averages ${Z}_{1}={\delta }_{x}{\delta }_{y}\mathop{\sum }\nolimits_{i,\,j}^{n}{I}_{i,\,j}$ and ${Z}_{2}={\delta }_{x}{\delta }_{y}\mathop{\sum }\nolimits_{i,\,j}^{n}{S}_{i,\,j}$. Recall from Extended Data Fig. 1 that these variables alone are insufficient to determine their subsequent evolution. Our goal is to learn closure variable(s), and a stochastic dynamics that describes the evolution of these variables.

Training data are generated through integrating equation (22) using the Euler–Maruyama method with time step size d*t* = 0.03. The initial conditions are selected as ${I}_{i,\,j}(0)=5{{\mathrm{e}}}^{-{({x}_{i}-{x}_{0})}^{2}-{({y}_{j}-{y}_{0})}^{2}}$ and ${S}_{i,\,j}(0)=5{{\mathrm{e}}}^{-{({x}_{i}-1)}^{2}-{(\,{y}_{j}-1)}^{2}}+5{{\mathrm{e}}}^{-{(x_{i}-1)}^{2}-{(\,y_{j}+2)}^{2}}$, where *x*_0_ and *y*_0_ are randomly generated from a uniform distribution in the unit square, and *x*_*i*_ = −5 + *iδ*_*x*_ and *y*_*j*_ = −5 + *jδ*_*y*_. This initial condition corresponds to the scenario where the initial susceptible population is fixed, but the initial infective population is a cluster that is uniformly and randomly distributed in the domain. Then we carry out the S-OnsagerNet workflow as shown in Fig. 1, with *β* = 0.01, *α* = 0.001, *m* = 50 and *d* = 3 (that is, one closure coordinate). We employ a PCA-encoder network to obtain the closure coordinate. The training process involves initially training the PCA-encoder and the S-OnsagerNet simultaneously. Subsequently, we fix the former and continue training the latter to a desired error tolerance. Note that in the SIR model case, we do not utilize the decoder network as there is no need to obtain a reconstruction of *I* and *S*.

#### Capturing the stochastic dynamics

First, we show that with just one additional learned closure coordinate *Z*_3_, we can capture the statistics of the macroscopic dynamics of the spatial averages of infective and susceptible individuals. The results are shown in Extended Data Fig. 1d,e, where the true mean and standard deviation of *Z*_1_ and *Z*_2_ are obtained from equation (22), while the predicted results are derived from S-OnsagerNet. Four representative test initial conditions are shown: two with disease spread and the other two without. We observe that we can successfully capture the macroscopic dynamics of disease spread with just one additional closure coordinate.

#### Interpreting the closure coordinate

Next, as we have shown that only one closure coordinate is required for a thermodynamic description, it is natural to probe its physical meaning. We use the same technique described in the polymer stretching case, where we investigate the effect of perturbation of a microscopic state in the directions that cause the sharpest changes in *Z*_3_. The microscopic state we probe is chosen as a pair of partially overlapping clusters of infective and susceptible individuals (Extended Data Fig. 2a). We observe that the perturbations induced by d*Z*_3_/d***X*** (where *X* = (*I*, *S*)) correspond to increasing/decreasing the overlap of clusters of susceptible and infected individuals. Hence, this suggests that *Z*_3_ is a macroscopic descriptor that correlates with such effective spatial overlap. We confirm this hypothesis via a scatterplot in Extended Data Fig. 2b, where the overlap is defined by $I{S}_{{\mathrm{mean}}}={\delta }_{x}{\delta }_{y}\mathop{\sum }\nolimits_{i,\,j}^{n}{I}_{i,\,j}{S}_{i,\,j}$. Thus, we can interpret the closure coordinate *Z*_3_ as an indicator of the overlap of clusters of susceptible and infective individuals. This is physically sensible, as the measure of overlap of spatial clusters should determine the outcome of an epidemic. Nevertheless, we must emphasize that the learned *Z*_3_ is a quantitative measure and can be applied to more complex configurations than a pair of clusters, for which one may not be able to easily define a notion of effective overlap by empirical observation.

#### Free-energy landscape

Finally, we study the dynamical landscape of the learned S-OnsagerNet model. Using the same projection technique in the polymer case, we plot two-dimensional projections of the learned three-dimensional free-energy landscape in Extended Data Fig. 3, overlaid with two representative trajectories (with disease spread in blue and disease dying out in red).

A number of interesting features can be gleaned from the landscape. First, we can clearly see the origins of the divergence of the two different types of trajectory. While they have identical initial *Z*_1_ (average infective) and *Z*_2_ (average susceptible) values, their initial *Z*_3_ (infective/susceptible overlap) values differ (Extended Data Fig. 3b,c). In particular, the initial disease spread seen in the red and blue trajectories is approximately in accordance to the steepest descent of the energy landscape. That is, the initial *Z*_3_ value is a determining factor for the onset of epidemics. Second, we compute using the steady states of the dynamics corresponding by solving ∇*V*(***Z***) = 0. Instead of isolated steady states as in the case of polymer dynamics, we find a one-dimensional manifold of stable steady states in the *Z*_2_–*Z*_3_ plane (Extended Data Fig. 3c,f). This implies, in particular, that the terminal state (the remaining number of susceptible individuals) is not unique, but rather depends on the initial configuration, and in particular on the initial overlap described by the learned coordinate *Z*_3_. This rationalizes the observed heterogeneity in the terminal configurations as shown in Extended Data Fig. 1.

### Statistics and reproducibility

The train–test splits in this paper are performed by random uniform subsampling. Repeats of numerical experiments are performed by running the same code with different random seeds. No data were excluded from the analyses, and the investigators were not blinded to allocation during experiments and outcome assessment.

### Reporting summary

Further information on research design is available in the Nature Portfolio Reporting Summary linked to this article.

### Supplementary information

Supplementary Information Supplementary Figs. 1–11 and Tables 1 and 2.

Reporting Summary

Supplementary Video 1 Evolution of a slow and fast stretching trajectory and their reduced coordinates overlaid on the learned free-energy landscape.

### Source data

Source Data Fig. 2 Source data for trajectory plots.

Source Data Fig. 3 Source data for trajectory plots.

Source Data Fig. 4 Source data for free-energy landscape, trajectory plots, location of steady states and so on.

Source Data Fig. 5 Source data for uncontrolled and controlled trajectories.

Source Data Extended Data Fig. 1 Source data for trajectory plots.

Source Data Extended Data Fig. 2 Source data for configuration and correlation plot.

Source Data Extended Data Fig. 3 Source data for trajectory and free-energy landscape.

## Data Availability

The simulation and experimental datasets used are publicly available in the Harvard Dataverse public repository^51^. The simulation data were generated according to the methods introduced in ‘Data preparation’ in Methods. Source data are provided with this paper.

## Code availability

The code to reproduce the analysis generated within the study is provided at https://github.com/MLDS-NUS/DeepLearningCustomThermodynamics ref. ^52^.
