# Supplementary material for: Constructing custom thermodynamics using deep learning
Source: Nat Comput Sci. 2023 Dec 29;4(1):66–85. doi: 10.1038/s43588-023-00581-5 (PMC10808076; doi:10.1038/s43588-023-00581-5)
Supplement: Supplementary file 1 — Supplementary Figs. 1–11 and Tables 1 and 2. [file 43588_2023_581_MOESM1_ESM.pdf]

nature computational science

Article https://doi.org/10.1038/s43588-023-00581-5

Constructing custom thermodynamics using deep learning

In the format provided by the authors and unedited

Contents

1 Supplementary figures **2**

2 Supplementary tables **8**

1 Supplementary figures

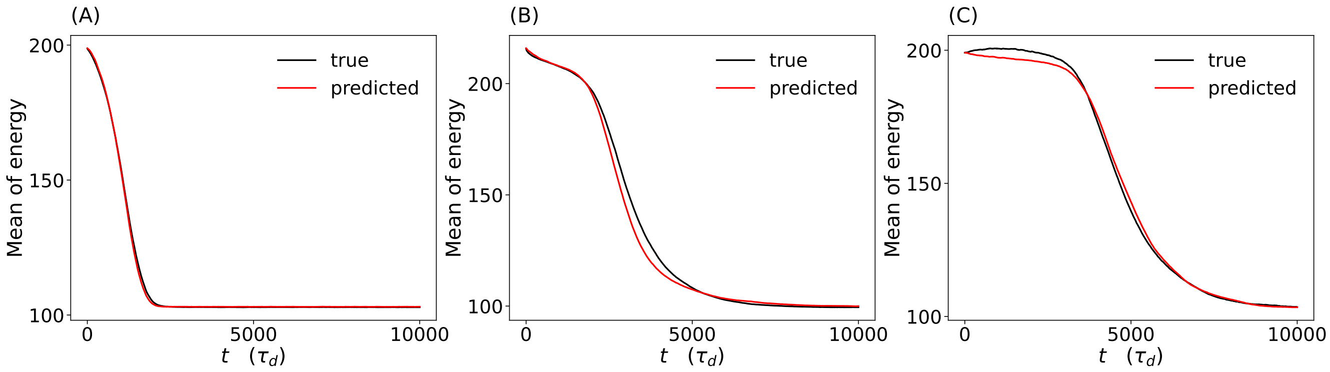

Supplementary Figure 1: **The time evolution of the mean of the energy V for polymer dynamics.** (A) fast trajectory; (B) medium trajectory; (C) slow trajectory.

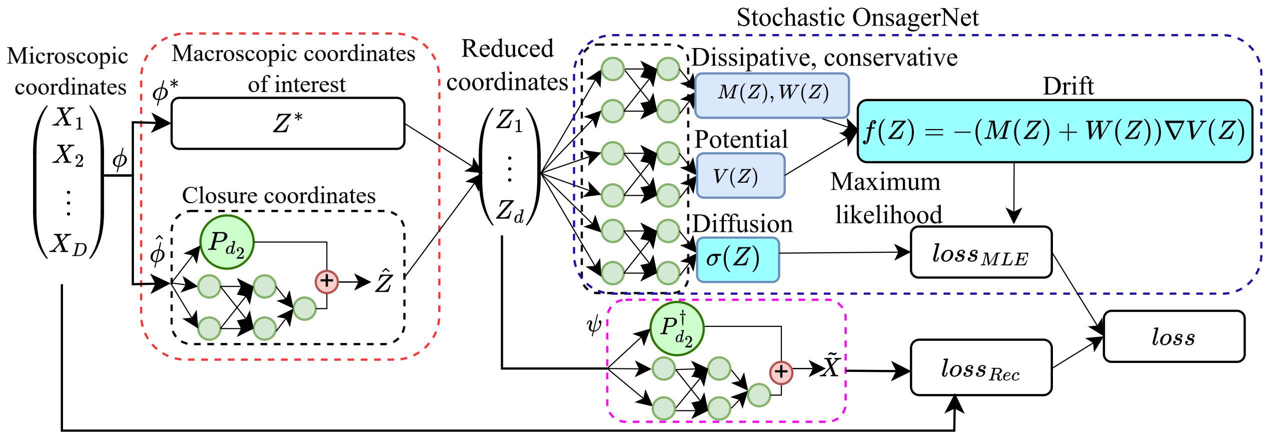

Supplementary Figure 2: **Detailed S-OnsagerNet workflow.** The input data $X(t) = (X_1(t), \cdots, X_D(t))^T \in \mathbb{R}^D$ are the microscopic coordinates. The red box contains the components that discovers reduced coordinates $Z(t) = (Z^*(t), \hat{Z}(t))^T = (Z_1(t), \cdots, Z_d(t))^T \in \mathbb{R}^d$, where ϕ^* is known, and $\hat{\phi}$ is PCA-ResNet with P_d the PCA projection matrix (to the first $d-1$ principal components). The blue box encloses the main S-OnsagerNet architecture to learn the low dimensional stochastic dynamical system. The function ψ is a decoder neural network with output $\tilde{X}$, where $P_d^\dagger$ is the pseudo-inverse of P_d. The reconstruction error $\mathrm{loss}_{\mathrm{Rec}}$ and the maximum likelihood loss $\mathrm{loss}_{\mathrm{MLE}}$ are combined to obtain the total loss

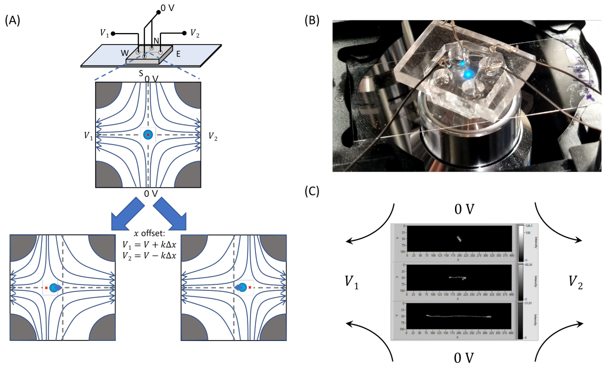

Supplementary Figure 3: **Schematic of experimental setup.** (A) Top: Schematic of the experimental setup, consisting of a microfluidic cross-slot device and electrodes in the North, South, East and West reservoirs. V_1 and V_2 are computer-controlled voltages based on a feedback control system. Center: Negatively charged DNA molecules flow through the cross-slot channel according to the electric field lines. The blue circle represents an object at the saddle point. Bottom left, right: A proportional gain controller is used to trap and stretch a DNA molecule at the saddle point for long observation times in a planar elongational field. (B) Photo of microfluidic device sitting atop the microscope stage and arrangement of electrodes in the reservoirs. (C) Snapshots of a DNA molecule stretching under an elongational field.

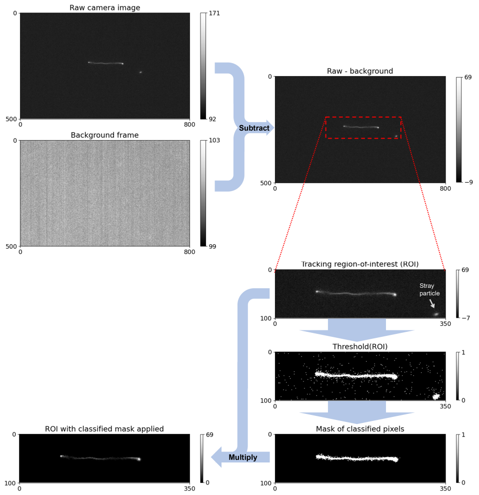

Supplementary Figure 4: **Data filtering process for experimental images.** Image processing pipeline, showing the steps for obtaining a clean molecule image from a raw camera frame in real time. The clean image (bottom-left) is used to calculate molecule centroid coordinates and projection lengths in both axes.

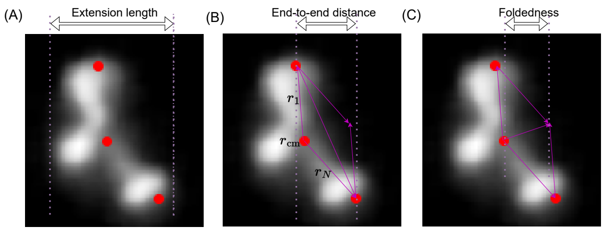

Supplementary Figure 5: **Obtaining extension length (A), end-to-end distance (B) and foldedness (C) from experimental image with center of mass r_{cm}, and two end points.**

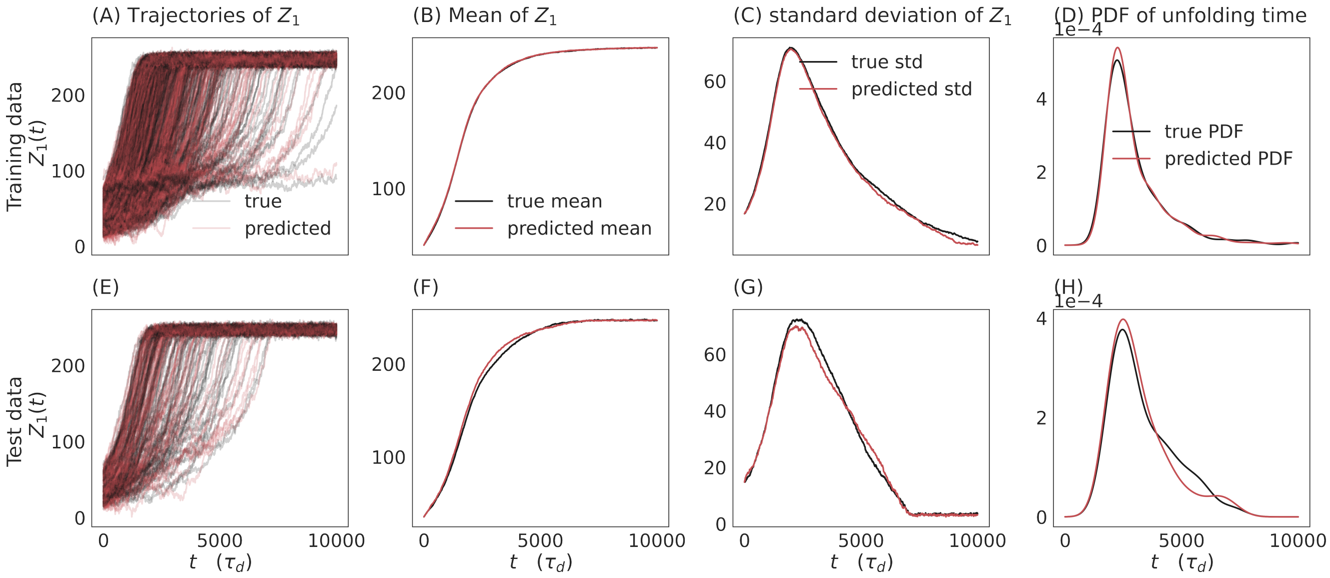

Supplementary Figure 6: **Predicted stretching trajectories and statistics for 610 training trajectories (up) and 110 test trajectories (down): true data (black) and model prediction (red).** (A,E) Individual stretching trajectories of polymer chains from the different initial condition. (B,F) Mean and (C,G) standard deviations of polymer chain extensions. (D,H) Probability density function (PDF) of the chain unfolding times.

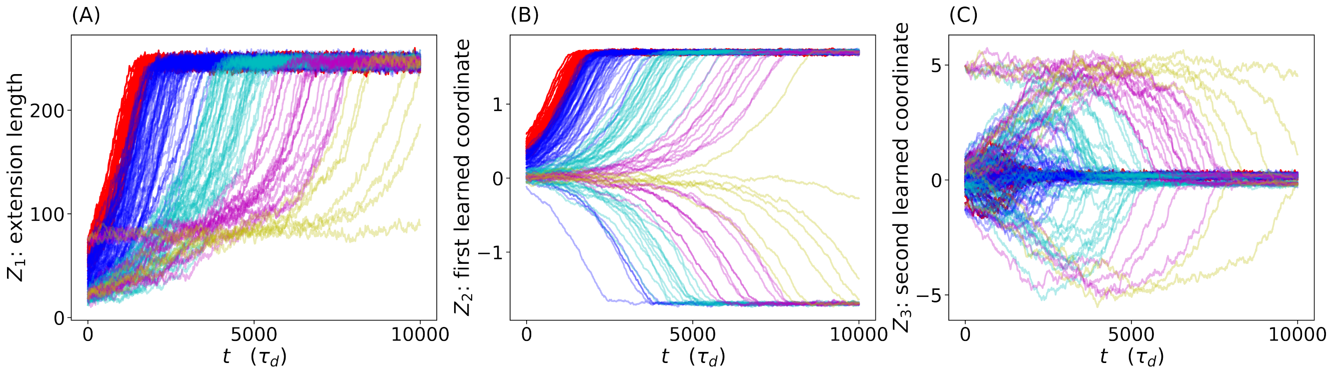

Supplementary Figure 7: **Learned reduced coordinates.** Evolution of (A) chain extension (Z_1), (B) first learned coordinate (Z_2, indicator of end-to-end distance) and (C) second learned coordinate (Z_3, indicator of foldedness) with time. The trajectories are colored by the chain unfolding times, red: $t_{\text{unfold}} < 2000$; blue: $2000 \leq t_{\text{unfold}} < 4000$; cyan: $4000 \leq t_{\text{unfold}} < 6000$; magenta: $6000 \leq t_{\text{unfold}} < 8000$; yellow: $t_{\text{unfold}} \geq 8000$.

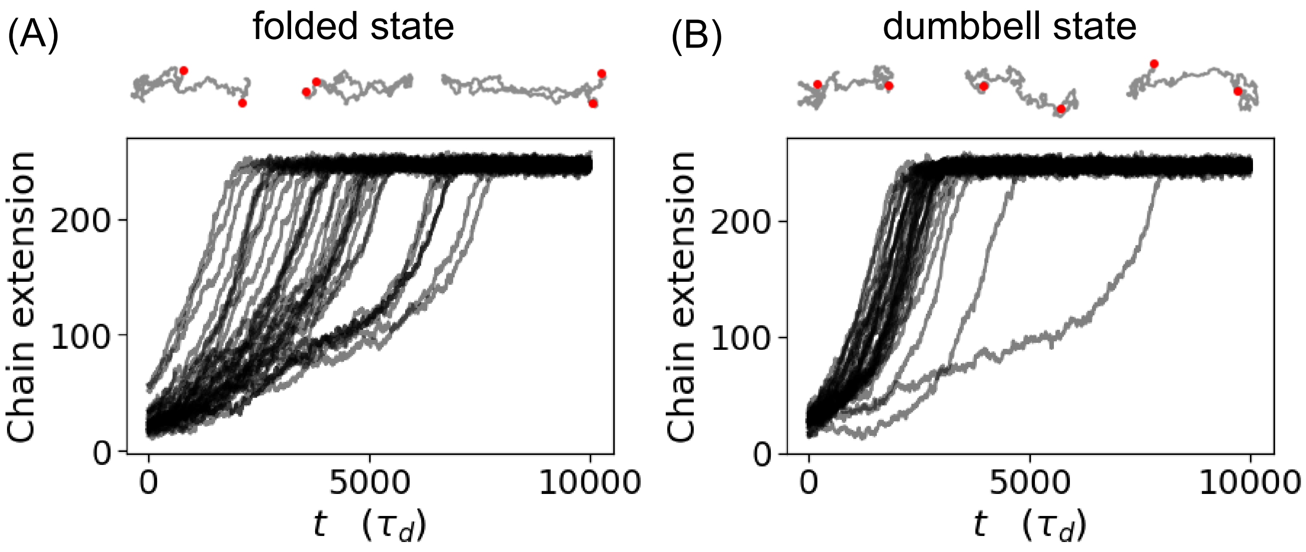

Supplementary Figure 8: **Stretching trajectories for polymer chains in the (A) folded and (B) dumbbell states.** The chain configurations were identified as described by Perkins et al. There is a large range of unfolding times within a given configuration type, hence the classification of chain configuration is insufficient for prediction purposes.

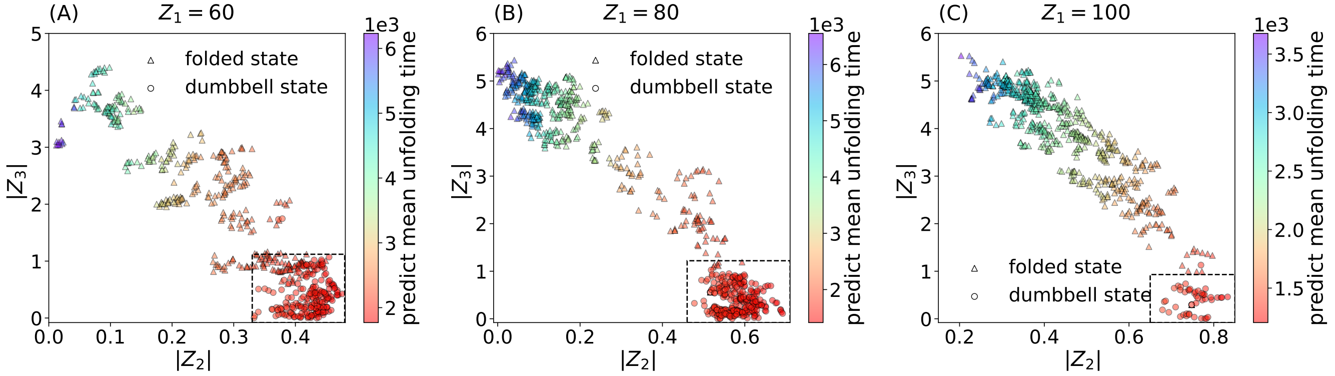

Supplementary Figure 9: **Consistency with configuration categorization scheme in current literature.** Plot of $|Z_3|$ as a function of $|Z_2|$ for folded and dumbbell configurations at (A) $Z_1 = 60$, (B) $Z_1 = 80$ and (C) $Z_1 = 100$. The markers are colored by the predicted chain unfolding times. The boxed region with high $|Z_2|$ and low $|Z_3|$ values encompasses a mix of folded and dumbbell chains with similar unfolding times, indicating that the broad categorization scheme is unable to provide accurate predictions.

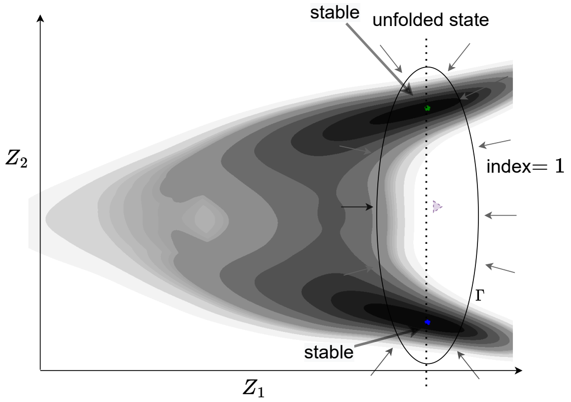

Supplementary Figure 10: **Illustration of the limitation of a 2-dimensional potential landscape.** Due to the stability of the unfolded state, the vector fields around the curve Γ point inwards towards the interior, so the index of Γ is $+1$. However, this contradicts the presence of two stable critical points inside Γ, which implies that its index is $+2$.

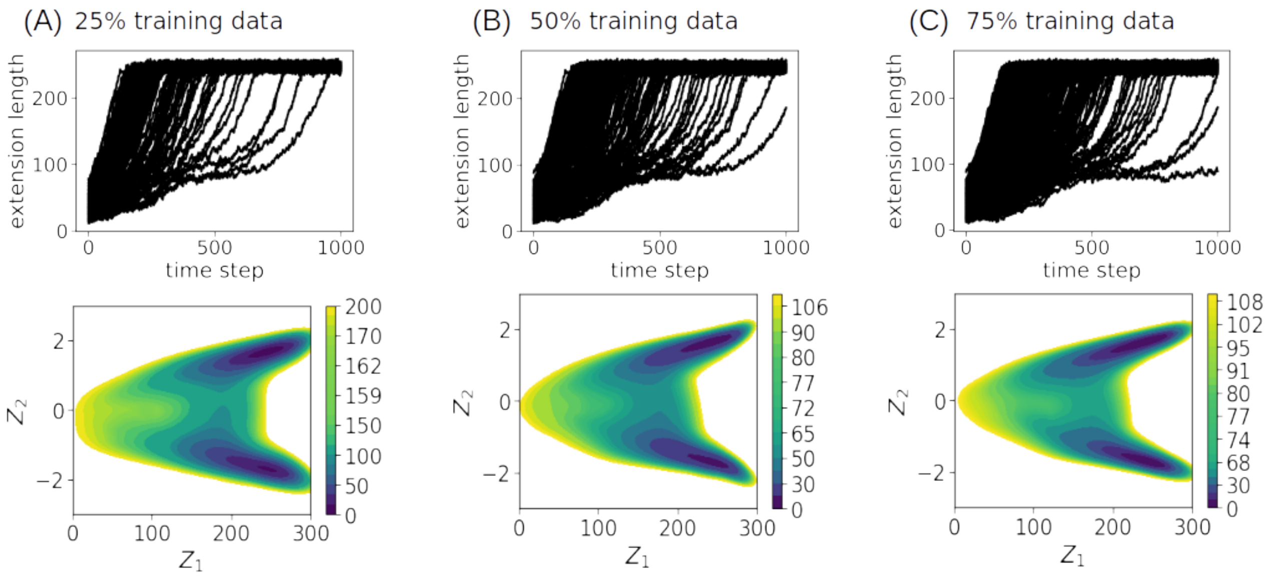

Supplementary Figure 11: **Trajectories and potential landscapes for different percentages of training data.** The full dataset, as used in the rest of this work, contains 610 trajectories. In order to evaluate the impact of dataset size on prediction results, the S-OnsagerNet was trained using (A) 25%, (B) 50% and (C) 75% of the 610 trajectories. The datasets in (A) and (C) (top) do not have shared trajectories, whilst the dataset (B) (top) contains data from both the 25% and 75% datasets. The potential landscapes (bottom) resulting from training with the different number of trajectories are plotted for Z_1 vs Z_2. It can be observed that each contains the characteristic features of the potential landscape discussed for the full dataset, with two areas of near-zero potential when the DNA reaches the steady-state, and the emergence of a saddle point around $Z_1 \approx 110$.

2 Supplementary tables

Supplementary Table 1: Variation in prediction error with number of reduced dimensions. Note that the dimension here includes the chosen macroscopic coordinate corresponding to polymer extension length.

relative L^2 error of training/test data (%)			
Dimension	mean	standard derivation	PDF of unfolding time
2D	0.7927/2.086	13.40/32.96	12.63/17.43
3D	0.2828/1.861	3.147/6.057	5.717/12.42
4D	0.4363/1.363	9.329/30.62	3.041/10.52

Supplementary Table 2: Variation in test prediction error of 3D model with the number of trajectories in the training data. We group the test trajectories into three categories (fast, medium, slow) according to their rate of stretching.

relative test L^2 error of mean/ standard derivation /pdf of unfolding time (%)			
Number of trajectories	fast	medium	slow
153 (25%)	0.246/11.38/26.37	6.749/27.51/44.80	9.666/55.33/33.81
305 (50%)	2.504/23.52/72.49	2.967/12.03/19.63	3.404/8.438/14.06
457 (75%)	1.206/16.91/26.34	6.581/35.28/34.80	2.120/8.866/15.56
610 (100 %)	0.388 /13.06/15.95	2.101/9.227/20.18	2.027/7.121/13.42
